# Mitochondrial introgression hampers the DNA barcoding of cryptic yellow fever vectors *Haemagogus capricornii* Lutz and *Hg. janthinomys* in the Atlantic Forest, Brazil

**DOI:** 10.1186/s13071-026-07343-y

**Published:** 2026-05-11

**Authors:** Filipe Vieira Santos de Abreu, Lucas Bonato Mosmann, Carolina Boucinha Martins, Alexandre da Silva Xavier, Igor Mello da Rocha Corpas Maciel, Agostinho Cardoso Nascimento-Pereira, Paulino Siqueira Ribeiro, Jeronimo Alencar, Rosa Maria Tubaki, Monique Albuquerque Motta, Ricardo Lourenço-de-Oliveira, Márcio Galvão Pavan

**Affiliations:** 1 Laboratório de Mosquitos Transmissores de Hematozoários, Instituto Oswaldo Cruz, FIOCRUZ, Rio de Janeiro, RJ Brazil; 2 Laboratório de Comportamento de Insetos, Instituto Federal do Norte de Minas Gerais, Salinas, MG Brazil; 3 Laboratório de Diptera, Instituto Oswaldo Cruz, FIOCRUZ, Rio de Janeiro, RJ Brazil; 4 Programa de Pós-Graduação em Biologia Parasitária, Universidade Federal de Sergipe, São Cristóvão, SE Brazil; 5 Instituto Pasteur, São Paulo, SP Brazil

**Keywords:** *Haemagogus*, Integrative taxonomy, Yellow fever, Speciation, DNA barcoding, Mito-nuclear phylogenetic discordance, Introgression, Incomplete lineage sorting, Pleistocene

## Abstract

**Background:**

Yellow fever is a major public health concern in Brazil, transmitted in sylvatic cycles by *Haemagogus* and *Sabethes* mosquitoes. Among them, *Haemagogus janthinomys* and *Hg. capricornii* occur in sympatry in the Atlantic Forest and females are morphologically indistinguishable, complicating vector identification during outbreaks. Here, we aimed to investigate their taxonomic status and evolutionary history using an integrative approach including morphological and phylogenetic analyses.

**Methods:**

Mosquitoes were collected in 17 municipalities across nine Brazilian states, including simultaneous captures of both species in sympatric areas. Males were identified by genitalia morphology and molecular analyses were performed using three mitochondrial and two nuclear genes. Diversity analyses and neutrality tests were performed, and phylogenies were reconstructed with Maximum Likelihood and Bayesian inferences. Divergence times were estimated using strict molecular clock, and population history was assessed through mismatch distribution analysis and Bayesian Skyline Plots.

**Results:**

A total of 79 specimens were morphologically identified, with *Hg. janthinomys* showing a broader geographic and altitudinal distribution than *Hg. capricornii*, which was usually restricted to higher elevations. Phylogenetic analyses based on mitochondrial markers revealed two clades, but did not recover clear reciprocal monophyly, thus evidencing that these markers alone cannot separate the two species. The inclusion of nuclear markers evidenced introgression events of *Hg. janthinomys* mitochondria in *Hg. capricornii* specimens in the Paraíba River Valley and Espírito Santo State, and successive breeding of *Hg. capricornii* on few samples morphologically identified as *Hg. janthinomys* in São Paulo State. Molecular clock and population history analyses evidenced that these species have probably speciated in peripatry or parapatry during the Pleistocene era at approximately 1.2 million years ago, and a recent sudden expansion of *Hg. capricornii* in the last 10 thousand years ago has tripled its population and likely led to a secondary contact between the two species.

**Conclusions:**

*Haemagogus janthinomys* and *Hg. capricornii* are valid and closely related species with evolutionary histories shaped by divergence during the Pleistocene era and subsequent introgression events. The use of cytochrome c oxidase subunit I gene (COI) DNA barcoding alone could not reliably distinguish them, and integrating morphology with multiple molecular markers is essential for accurate identification. Future work is needed for a finer resolution of hybridization patterns to help clarify if the observed mito-nuclear discordance reflects historical introgression or active genetic exchange between species.

**Graphical abstract:**

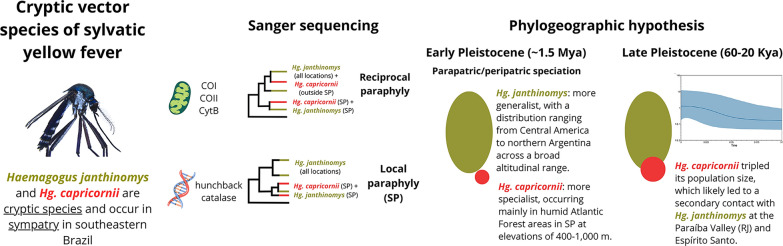

**Supplementary Information:**

The online version contains supplementary material available at 10.1186/s13071-026-07343-y.

## Background

Yellow fever virus (YFV; *Flaviviridae*: *Orthoflavivirus flavi*) is the etiological agent of yellow fever (YF), an acute febrile disease of varying severity in humans, with a fatality rate ranging from 20 to 60% of cases [1]. On the American continent, YFV is transmitted in a sylvatic cycle by mosquitoes of the genera *Haemagogus* Williston and *Sabethes* Robineau-Desvoid (Diptera: Culicidae). In Brazil, YFV circulates perennially in the Amazon region and, in recent decades, has caused sporadic outbreaks in the extra-Amazon area. The largest YF outbreak occurred between 2014 and 2021, with the peak of human cases recorded around 2017–2019 (1271 cases and 719 deaths), when the virus reached southeastern Brazil, mostly covered by the Atlantic Forest [2, 3]. Together with *Haemagogus leucocelaenus* (Dyar and Shannon, 1924) [4], entomological investigations during this outbreak incriminated *Hg. janthinomys* (Dyar, 1921) [5] or *Hg. capricornii* Lutz, 1904 [6] as the primary vectors [7–10]. Female mosquitoes are by far the main gender captured during outbreak investigation samplings. Nevertheless, adult females of *Hg. janthinomys* and *Hg. capricornii* are morphologically identical, which prevented specific identification and determination of the role of each species in the YFV transmission in the aforementioned outbreak [7–10].

*Haemagogus janthinomys* was described from specimens collected in Port of Spain, Trinidad and Tobago, and occurs in a wide geographic area from Central America to southern Brazil [5, 11]. In the Atlantic Forest, however, the distribution of *Hg. janthinomys* partially overlaps with that of *Hg. capricornii,* a sibling species described by Lutz in 1904 from adults reared from eggs collected in Cantareira, a remnant of the Atlantic Forest of São Paulo, southeastern Brazil. While *Hg. janthinomys* is distributed across distinct biomes such as the Amazon, Cerrado, and Atlantic Forest, *Hg. capricornii* is predominantly restricted to the Atlantic Forest of southern, southeastern, and northeastern Brazil [11, 12].

Historically, these two species were considered synonyms, until Arnell [11] morphologically characterized them as distinct species on the basis of the male genitalia characters. No morphological difference has been found in the females, larval, and pupal stages. To date, the only way to safely differentiate the two species is by analyzing the male genitalia morphology, specifically in the aedeagus and the apex of paraproct [11]. Hence, the development of new tools is required to facilitate the taxonomic differentiation of these two morphotypes. An integrated approach between morphological and molecular taxonomy would contribute greatly to solving taxonomic gaps among these sibling species and understand their genetic relationships and evolution.

In recent years, molecular markers such as mitochondrial cytochrome c oxidase subunit I gene (COI) among other genes have been contributing to taxonomic and phylogenetic studies of several groups, including mosquitoes [13, 14]. In addition, the complete mitochondrial genome of *Hg. janthinomys* and other congeneric species have been recently revealed, which ended up confirming and reinforcing the phylogeny originally proposed through morphological taxonomy [15, 16]. However, molecular markers were rarely used to distinguish *Hg. janthinomys* from *Hg. capricornii*. To our knowledge, studies published to date have relied on a single molecular marker (COI) to distinguish the two species, on the basis of samples collected from a single state in southeastern Brazil, without independent confirmation of the morphological identification of the sequenced specimens [17, 18]. The present work aimed to present a phylogenetic analysis of these nominal species using both mitochondrial and nuclear markers as well as morphological characters of specimens collected in different locations in Brazil, including simultaneous captures in sympatric areas.

## Methods

### Mosquito collection and rearing

**Fig. 1:**
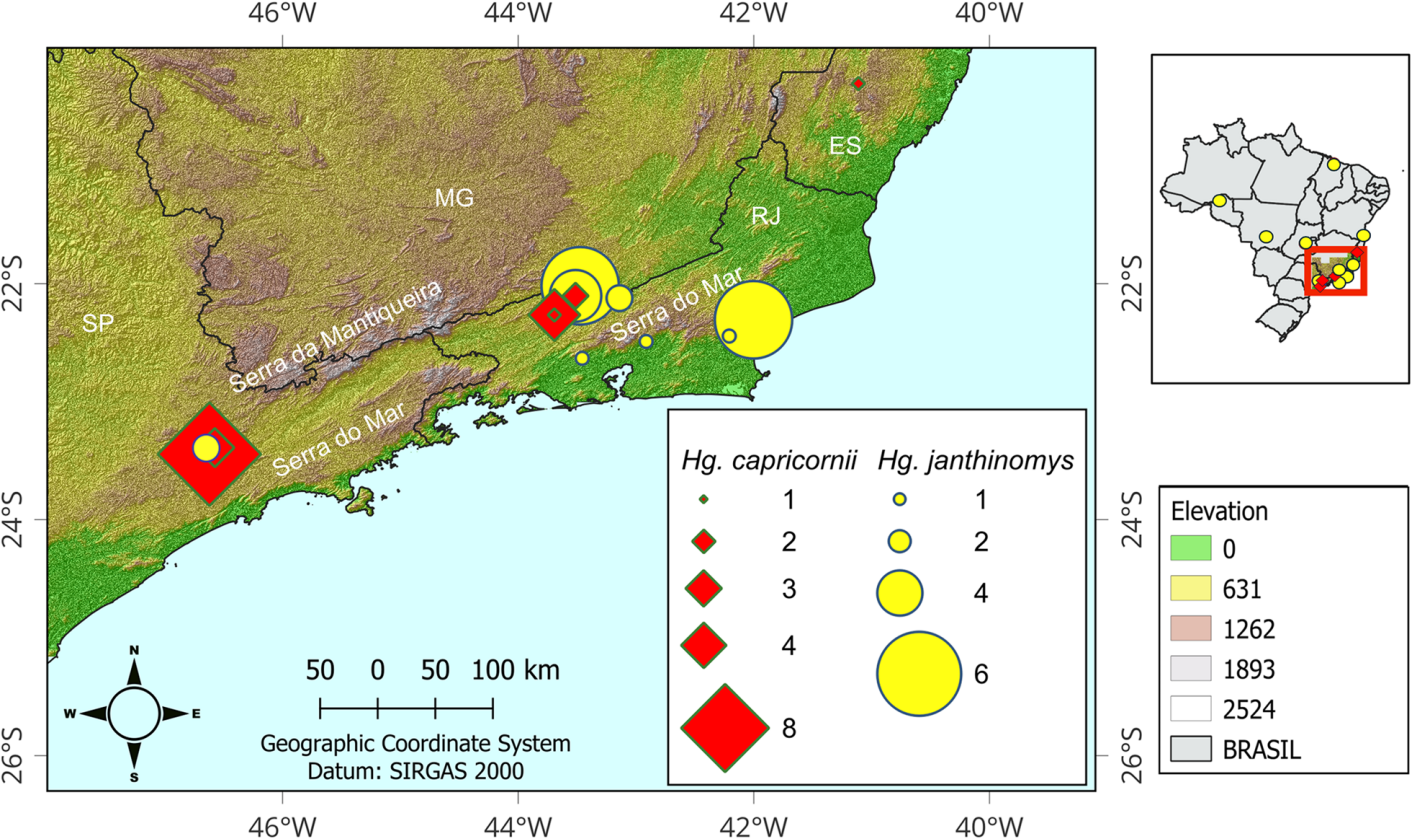
Collection sites of *Hg. janthinomys* and *Hg. capricornii* in southeastern Brazil and their respective altitudes. Brazilian states: ES (Espírito Santo), MG (Minas Gerais), RJ (Rio de Janeiro), and SP (São Paulo)

The specimens used in the present study were collected in 17 municipalities distributed in nine Brazilian states: Bahia—BA, Maranhão—MA, Rondônia—RO, Goiás—GO, Mato Grosso—MT, Minas Gerais—MG, Espírito Santo—ES, Rio de Janeiro – RJ, and São Paulo—SP. The surveyed municipalities in the last four states were supposed to be *Hg. janthinomys* and *Hg. capricornii* sympatric areas, and the type locality of *Hg. capricornii* (Horto Florestal da Cantareira, SP) was included (Fig. 1, Additional file 1: Supplementary Tables S1 and S2, Additional file 2: Supplementary Fig. S1). Samplings occurred between September 2016 and March 2019, including the 2017–2019 YFV outbreak in southeastern Brazil.

**Fig. 2:**
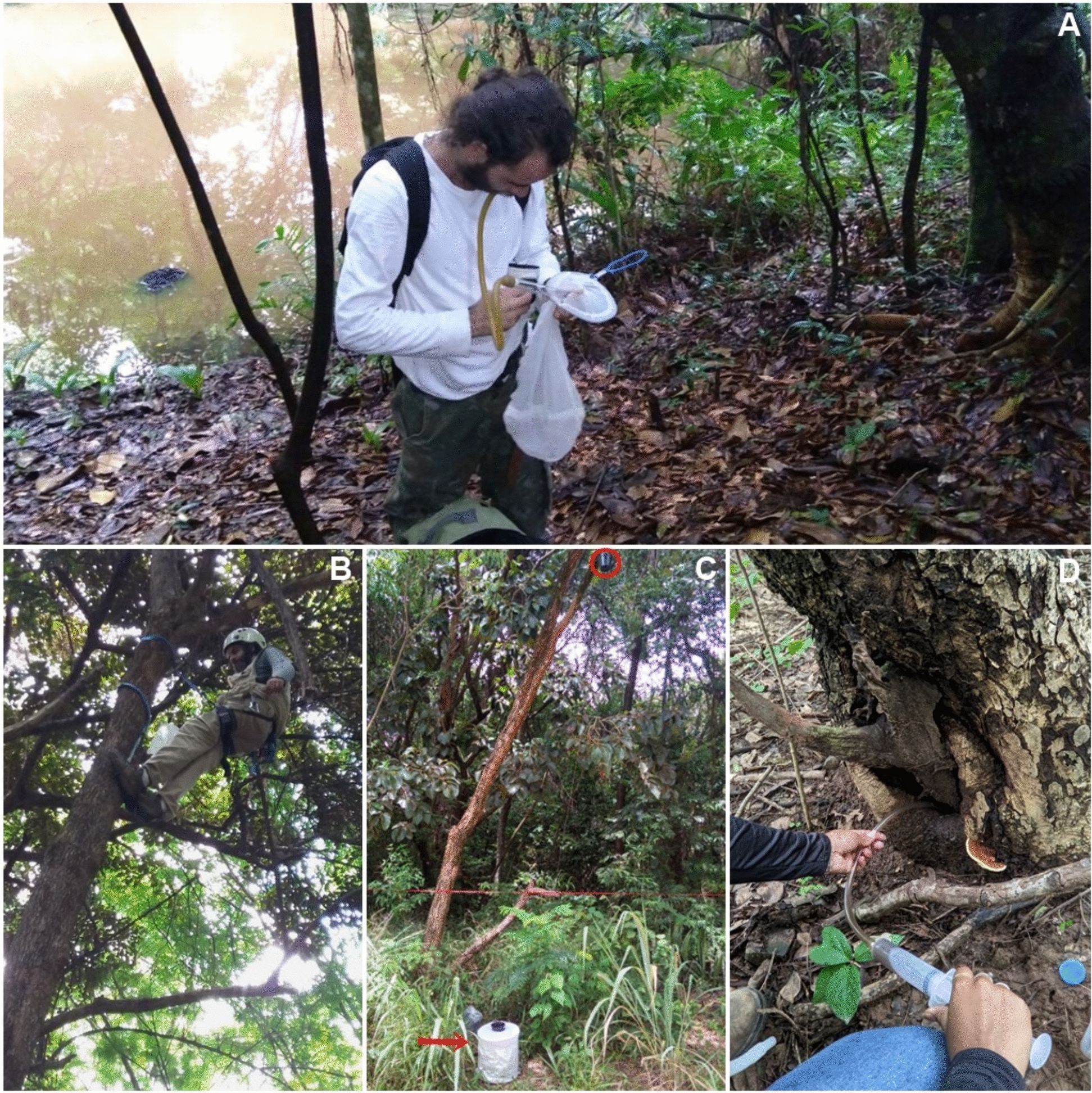
Sampling methods used to collect *Haemagogus* mosquitoes. Adults were collected through entomological net and oral aspirator at ground (**A**), and canopy levels (**B**), as well as through CO_2_ baited BG-sentinel on the ground (**C**, red arrow). Immatures were sampled using ovitraps suspended in the tree canopy (**C**, red circle) and by aspiration of water accumulated in natural breeding-sites (**D**)

Adults: The collection of adult mosquitoes occurred in forest fragments using different techniques: human attraction on the ground and at tree canopy levels, using nets and oral aspirators, and BG-sentinel traps baited with dry ice installed at ground level (Fig. 2A–C). The males, when collected with nets, were cryopreserved for later morphological identification and DNA sequencing. Females were transferred alive to the laboratory, blood-fed on mice, and placed in numbered individual tubes containing moistened cotton covered with filter paper on the bottom as support for oviposition. The papers containing the eggs of a single female were individually immersed several times in dechlorinated tap water supplemented with shed leaves in an insectary (26 ± 1 °C; 80 ± 10% of relative humidity; and 12 h:12 h light:dark cycles). The egg batches of each female underwent 10–20 cycles of at least 2 days of immersion and drying to stimulate hatching. The larval offspring of each female was raised together in pans containing dechlorinated tap water supplemented with yeast, which was renewed every 2–3 days. Fourth instar larvae were individualized until pupa formation and adult emergence. Adults of the same offspring were individually numbered and preserved at −80 °C for further morphological and molecular analysis. The Ethics Committee on Animal Use (CEUA) of Instituto Oswaldo Cruz approved mosquito feeding on mice (license no. LW-32/14).

Immatures: Larvae and pupae were collected in natural breeding sites (tree holes and cut bamboos) (Fig. 2D) and raised individually in the laboratory until adult emergence, as detailed above.

Eggs: Eggs were also obtained in the forests through ovitraps with usually three wooden paddles, as previously described [19]. The ovitraps were placed in the tree canopies at 3–5 m for 10–15 days (Fig. 2C). The positive paddles were taken to the laboratory, dried for 2–3 days in an insectary, and subsequently treated as described above for egg batches obtained on filter papers.

### Morphological identification

**Fig. 3:**
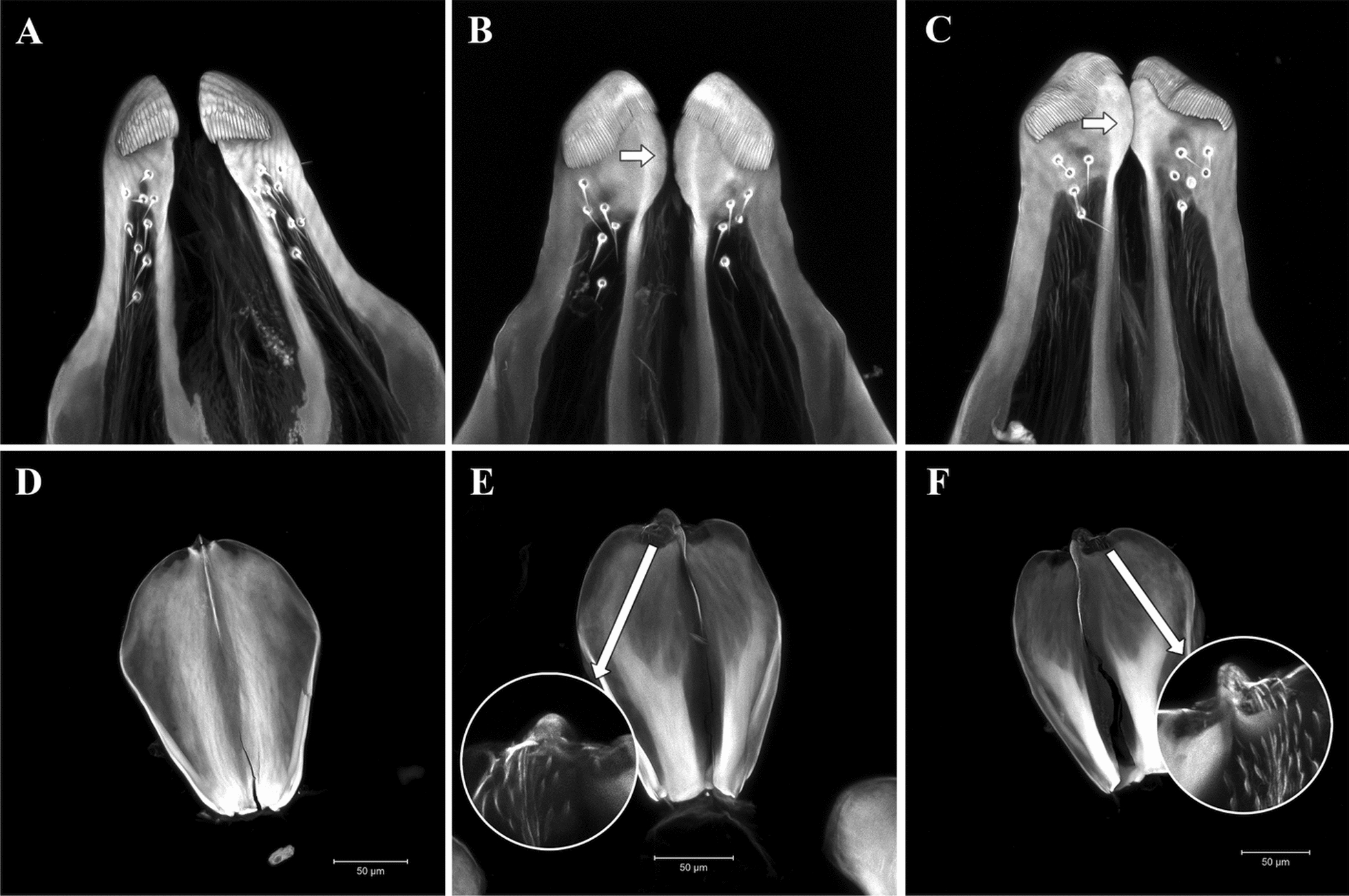
Male genitalia structures (paraproct and aedeagus) of *Haemagogus capricornii* (RF-186) and *Hg. janthinomys* (3R-108 and RF-169). **A** Paraproct of *Hg. capricornii* with straight apex. **B**, **C** Paraproct of *Hg. janthinomys* with arrow indicating rounded expansions the inner edge of the apex. **D** Aedeagus of *Hg. capricornii*, dorsal view, without spicules on upper mesal area. **E**, **F** Aedeagus of *Hg. janthinomys*, dorsal view, with spicules on upper medial area

All adult male mosquitoes obtained had their genitalia extracted, dissected, and mounted on slides associated with respective larval and pupal exuviae, and examined using an optical microscope. A sample of mounted male genitalia was additionally examined and photographed under a Zeiss LSM 710 confocal laser scanning microscope (Jena, Germany) at the Microscopy Core Facility of the PDTIS/FIOCRUZ, at Instituto Oswaldo Cruz, for detailed analysis of specific structures of aedeagus and paraproct. Images were subsequently processed through 3D reconstruction of serial z-stack images to render detailed anatomical visualization, following the protocol outlined by Ribeiro et al. [20]. Both male genitalia characters of the aedeagus and paraproct reported by Arnell [10] were used to differentiate the species: the presence of spicules on the upper distal mesal area of the aedeagus and apex of the paraproct with a hooklike process in *Hg. janthinomys* and a lack of these characteristics in *Hg. capricornii* (Fig. 3). All mounted specimens were cataloged and deposited in the *Coleção de Culicidae da Fiocruz* (Fiocruz-CCULI; Additional file 1: Supplementary Tables S1 and S2). The remaining parts of each mosquito were used for molecular analyses.

### DNA extraction, PCR, and DNA sequencing

Adult mosquitoes were placed individually into 2 mL microtubes with two 3.5 mm glass beads and grinded in the Tissue Lyser II (Qiagen, Hilden, Germany) for 1 min at 30 Hz. DNA was extracted from each sample with the Dneasy® Blood and Tissue Kit (Qiagen, Hilden, Germany) and quantified through fluorometry with the Qubit™ dsDNA HS Assay kit (Thermo Fisher Scientific, MA, USA), according to manufacturers’ protocols. Each sample was submitted to PCR amplification of three mitochondrial and two nuclear genes in a Veriti™ 96-Well Fast Thermal Cycler (Thermo Fisher Scientific, Massachusetts, USA), and had their amplified target DNA sequenced with the Sanger method in the PDTIS/FIOCRUZ genomic core facility at Instituto Oswaldo Cruz, Fiocruz (Rio de Janeiro, Brazil).

A 658-bp fragment of the mitochondrial cytochrome c oxidase subunit I (COI) gene, known as the “universal DNA barcoding fragment” for invertebrates [21] was PCR-amplified with the LCO1490/HCO2198 primers [22], following the conditions described in Ribeiro et al. [20]. Moreover, two pairs of primers were developed in this study to amplify a 593-bp fragment of the mitochondrial cytochrome c oxidase subunit II (COII), and a 550-bp fragment of the mitochondrial cytochrome b (CytB) (Additional file: Supplementary Table S3). Briefly, we retrieved mitochondrial genome sequences from GenBank (https://www.ncbi.nlm.nih.gov/genbank/) of the five *Haemagogus* (*Haemagogus*) species available—*Hg. spegazzinii* Brèthes, 1912 (NC057213), *Hg. janthinomys* (NC028025), *Hg. tropicalis* Cerqueira & Antunes, 1938 (NC057214), *Hg. albomaculatus* Theobald, 1903 (NC057211) and *Hg. equinus* Theobald, 1903 (PQ189399), and aligned the complete gene sequences in MEGA X [23]. Primer design was conducted *in silico* using Primer3Plus [24] to generate nucleotide pairs flanking target regions ranging from 400–800 base pairs, and primer quality was evaluated using the OligoAnalyser tool (https://www.idtdna.com/calc/analyzer) to check for melting temperature (Tm), potential secondary structure formation, and dimerization. Primers were considered adequate when (i) the Tm was above 45 °C, (ii) the difference between the Tm primers was less than 5 °C, (iii) and the Gibbs free energy of possible hairpin and dimers was less than −3 kcal/mol and −10 kcal/mol, respectively. We also amplified a 459-bp region of the first exon of the nuclear hunchback gene (hb) and a 690-bp region of the second exon of the nuclear catalase gene (cat), using the hbF/hbR and catF1/catR1 primers previously described [13].

Each 25-μL PCR reaction contained 2.5 μL 10 × High Fidelity Buffer [600 mM Tris-SO_4_ (pH 8.9), 180 mM (NH_4_)_2_SO_4_], 1.25 μL 2.5 mM dNTPs, 1.25 μL 50 mM MgSO_4_, 2μL of each primer at 10 μM, 2.5 U Platinum Taq DNA Polymerase (Invitrogen, Waltham, USA), and 20–30 ng of DNA. PCR amplification was performed in a Veriti^TM^ 96-Well Fast Thermal Cycler (Thermo Fisher Scientific, Massachusetts, USA), with the following thermal cycling profile for COII, hb, and cat: initial denaturation at 94 °C for 3 min; 35 cycles at 94 °C for 30 s, annealing temperature (Ta) for 1 min (Additional file 1: Supplementary Table S3), and 72 °C for 1 min; and a final extension at 72 °C for 10 min, which Ta were 55 °C, 57 °C, and 63 °C, respectively. The Touchdown-PCR strategy was used to amplify the CytB fragment, with an initial denaturation of 96 °C for 2 min; 15 cycles of 94 °C for 15 s, decreasing annealing temperature from 66 °C to 56 °C (0.5 °C every cycle) for 30 s, and 72 °C for 30 s; 10 cycles of 94 °C for 15 s, 63 °C for 30 s, 72 °C for 30 s, and final extension of 72 °C for 7 min.

Amplicons were purified with the GFX^TM^ PCR DNA and Gel Band Purification Kit (Cytiva, Marlborough, USA). Forward and reverse DNA strands were subjected to Sanger sequencing reactions, as previously described [20]. Capillary electrophoresis was run on an ABI 3730 automated sequencer (Applied Biosystems, CA, USA) in the RTP-01 core facility of the PDTIS/FIOCRUZ DNA Sequencing at Instituto Oswaldo Cruz. DNA electropherograms were visually inspected and edited whenever necessary in SeqMan Lasergene v. 7.0 (DNAStar, Inc., Wisconsin, USA). A consensus sequence was generated for each sample and for every molecular marker, and all were aligned with ortholog sequences with MAFFT 7.0 [25], using the L-INS-i algorithm. Other sequences of *Hg. janthinomys*, *Hg. capricornii*, and other *Haemagogus* (*Haemagogus*) spp. were retrieved from GenBank (Additional file 1: Supplementary Table S4).

### Genetic diversity and phylogenetic reconstructions

Saturation analysis was performed for COI sequences in R Studio [26], using the “ape” [27], “pegas” [28], and “ggplot2” [29] packages, owing to the large dataset comprising different *Haemagogus* species. This analysis compared Kimura’s two-parameter and *p*-distance models to understand the impact of multiple substitutions on evolutionary distance estimations. In case of saturation, it is expected to form a curved shape with a plateau, and when saturation is absent, a linear curve is observed.

Phylogenetic trees were inferred for each marker with both Maximum-Likelihood (ML) and Bayesian (BI) inferences, using IQ-Tree v.2 [30] and the BEAST 2.7 platform [31], respectively. For ML reconstructions, the best fit substitution model was obtained with ModelFinder, which calculates log-likelihoods on an initial parsimony tree and comparing models using Akaike and Bayesian Information Criteria (AIC and BIC), with the best-fitting model selected on the basis of the minimum BIC score [30]. Branch supports were determined with 1000 replicates of the SH-like approximate likelihood ratio test [32] for single branches, and 1000 replications of Ultrafast bootstrap. For BI, the best-fitting model of base substitution for each marker was selected using BIC in bModelTest 1.3 [33]. StarBeast3 v.1.1 [31, 34] was used to reconstruct Bayesian locus-specific phylogenetic trees with two independent runs with 5 × 10^7^ MCMC generations, using the coalescent model, and sampled parameters every 50,000 generations. The convergence of parameters and proper mixing of chains were evaluated in Tracer v. 1.7 [35] with the effective sample sizes (ESSs) were sufficiently large (ESSs > 10^4^). A strict molecular clock was imposed for all estimations of cladogenetic events, given that all analyses included closely related species belonging to the same genus and thus it is expected they evolved at a similar rate [36]. Two different rates were used in the phylogenetic reconstructions: for the mitochondrial markers, we assumed the divergence rate of 3.54% per million years (My; lognormal prior distribution with M = −4.035, S = 0.106, offset = 0, which gives a mean of 0.0177 and HPD 95% = 0.014–0.021), and about ten times slower divergence rate was imposed for nuclear markers (lognormal prior distribution with M = −6.5, S = 0.2, offset = 0, which gives a mean of 0.0015 and HPD 95% = 0.0010–0.0025) [37]. The confidence of clade reconstructions was inferred through posterior probabilities (PP).

Pairwise-sequence divergences were computed in MEGA X [23], using the Kimura 2-parameter model [38], with 500 bootstrap replicates to estimate standard errors, and a plot was generated using the ggplot2 [29], ggpubr [39], and ggrides [40] packages in R. Diversity indices, neutrality tests, and mismatch distribution analyses were performed in DNAsp v.6 [41]. Haplotype networks were created using the NETWORK v. 10.1 program (https://www.fluxus-engineering.com/sharenet.htm) with the median-joining method.

Since the COI was the largest dataset, it was used to infer the demographic history of *Hg. janthinomys* and *Hg. capricornii* using Bayesian Skyline Plot (BSP) analyses in BEAST 2.7 [31] under a strict molecular clock model, using the same divergence rate described above. Two independent runs were performed with 5 × 10^7^ MCMC generations, and parameters were sampled every 5000 generations. The convergence of parameters and proper mixing of chains were evaluated in Tracer v. 1.7 [35] with the effective sample sizes (ESSs) being sufficiently large (ESSs > 500). BSPs were also visualized in Tracer v1.7 [35].

## Results

### Morphological analysis

In total, 79 specimens were identified by examination of male genitalia, including 56 *Hg. janthinomys* and 23 *Hg. capricornii* (Fig. 1, Additional file 1: Supplementary Tables S1 and S2, Additional file 2: Supplementary Fig. S1). Additionally, two females were classified as *Hg. janthinomys* because they were siblings of morphologically identified males. As expected, specimens with morphotype corresponding to *Hg. janthinomys* had the widest distribution range, being found in seven states of North, Northeast, and Southeast regions (BA, MA, MG, MT, RJ, RO, and SP), while those matching with that of *Hg. capricornii* was found in only four states (ES, MG, RJ, and SP), all in the Southeast. Both morphotypes were found in sympatry in two sites located in RJ and SP, both belonging to the Atlantic Forest biome in Southeast region. Notably, *Hg. janthinomys* was recorded across a broader altitudinal range (19–920 m above sea level), whereas *Hg. capricornii* was restricted to higher elevations, occurring only between 447 and 1021 m above sea level (Additional file 1: Supplementary Table S1).

Besides the two diagnostic morphological characters reported by Arnell [11] to separate *Hg. janthinomys* and *Hg. capricornii*, we noted that the apex of the paraproct is almost straight in *Hg. capricornii* (Fig. 3A), while it has rounded mesal expansions in *Hg. janthinomys* (Fig. 3B, C). Male genitalia morphology was consistent within each progeny, with all male siblings exhibiting the diagnostic characters of either *Hg. capricornii* or *Hg. janthinomys* accordingly.

### Molecular analysis

#### Taxonomy

Molecular analysis included 63 specimens collected in this study (Additional file 1: Supplementary Table S1), and 88 additional sequences obtained from GenBank (Supplementary Table S4), and five genes were analyzed—three mitochondrial (COI, COII, and CytB) and two nuclear genes (*hunchback* and *catalase*). Among the 63 newly generated sequences, 62 individuals were morphologically identified to species level and one specimen (adult female) could not be identified beyond the genus level, being sequenced for COI, COII, and hunchback markers. Of the 88 GenBank sequences, 34 corresponded to specimens without definitive morphological identification. These specimens were herein labeled morphologically only as *Hg. janthinomys/capricornii*, but had species-level assignment through phylogenetic analyses based on COI sequences. Indeed, the majority of GenBank sequences were from the fragment of the COI gene (87 of 88), which was also the only marker available for *Haemagogus* species other than *Hg. janthinomys*, *Hg. capricornii*, and *Hg. spegazzinii*. Since no evidence of saturation was detected in the COI dataset (Additional file 2: Supplementary Fig. S2), the full *Haemagogus* sequence dataset, including third codon positions, was retained for subsequent analyses.

**Fig. 4:**
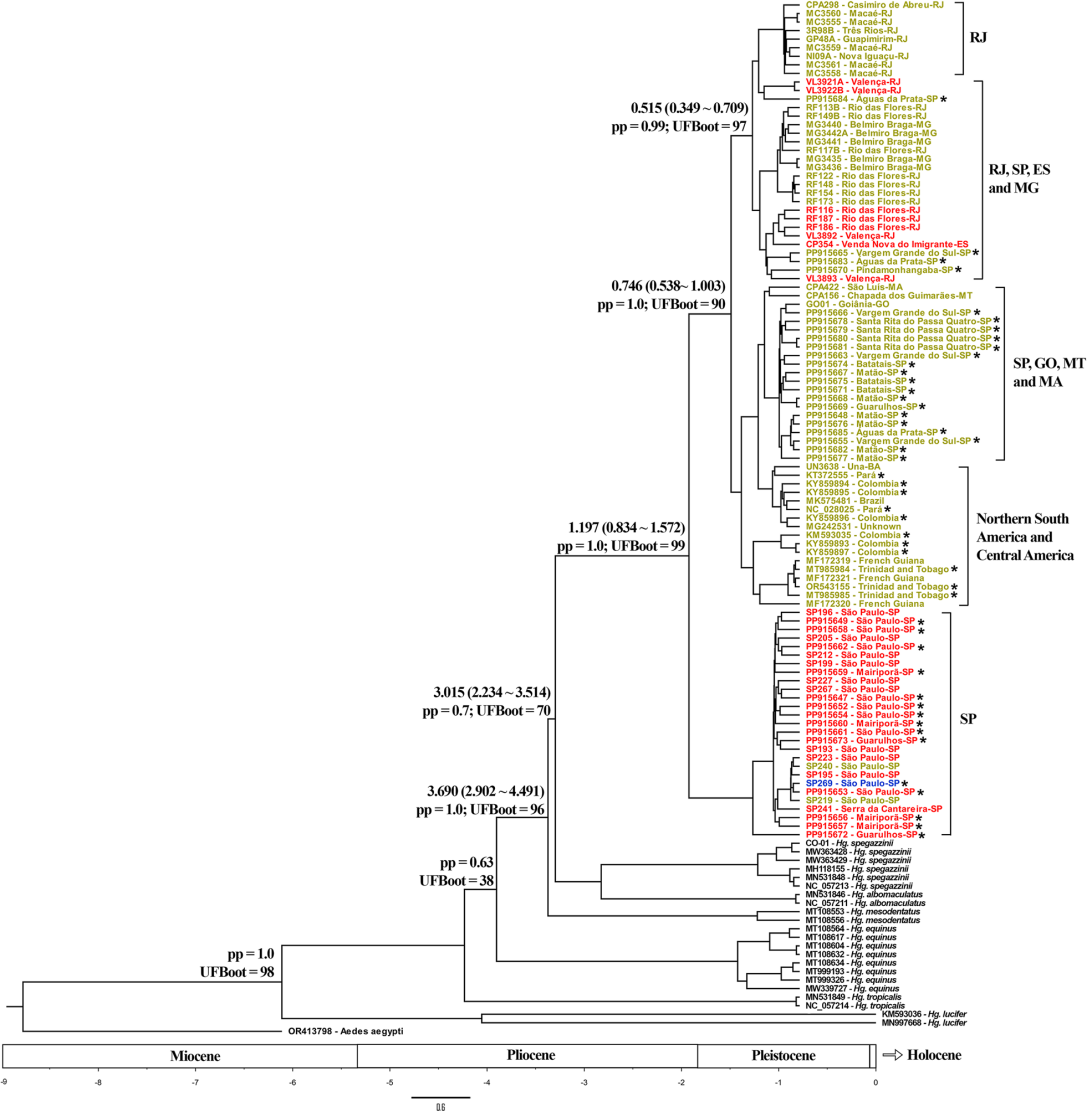
Bayesian phylogenetic reconstruction based on 658-bp of the mtCOI gene. Node labels indicate mean divergence times (in millions of years ago), with the confidence interval in parentheses, and also posterior probability (pp) and ultrafast bootstrap (UFBoot) values. Tip colors represent the morphological and/or previous molecular identification of specimens based on COI (according to Telles-de-Deus et al. [19]): dark yellow = *Hg. janthinomys*; red = *Hg. capricornii*; blue: female *Hg. janthinomys*/*Hg. capricornii*. Sequences of specimens with no information about the morphological identification at species level are marked with asterisks (*)

Bayesian phylogenetic reconstruction based on the COI gene of species of the *Haemagogus* subgenus (Fig. 4) revealed that *Hg. janthinomys* and *Hg. capricornii* were grouped into two major subclades separated from the remaining species (PP = 1.0, UFBoot = 99). *Haemagogus spegazzinii* and *Hg. albomaculatus* formed a moderately supported sister clade in relation to the *Hg. janthinomys/capricornii* clade (PP = 0.7, UFBoot = 70), and *Hg. mesodentatus* Komp and Kumm 1938 grouped as an external clade of these species (PP = 1.0, UFBoot = 96), followed by *Hg. equinus* (PP = 0.63, UFBoot = 38). Altogether, these species belong to the Albomaculatus Section, according to Arnell [11]. *Haemagogus tropicalis*, the single member of the Tropicalis Section, grouped closer to the Albomaculatus Section than *Hg. lucifer* (Howard, Dyar and Knab, 1912), which represented the Splendens Section, and was recovered as the most external species of the *Haemagogus* subgenus (PP = 1.0, UFBoot = 98).

Regarding the two clades comprising *Hg. janthinomys* and *Hg. capricornii* sequences, the first clade was more heterogeneous, and primarily composed of *Hg. janthinomys* sequences from eight different Brazilian states (BA, ES, GO, MA, MG, MT, PA, RJ, and SP; *n* = 54), and from Colombia, French Guiana, and sites in Trinidad and Tobago ranging from 5–10 km from the type-locality of this species (*n* = 12). Moreover, this clade also included eight sequences of all *Hg. capricornii* individuals collected outside the state of São Paulo (Valença and Rio das Flores, RJ; and Venda Nova do Imigrante, ES; Additional file 1: Supplementary Table S1). The second clade was more homogeneous and composed entirely of specimens from nearby cities of the state of São Paulo, in the metropolitan area (city of São Paulo, Guarulhos, and Mairiporã; *n* = 27). Most of the sequences clustered in this clade belonged to males morphologically identified as *Hg. capricornii*. Nevertheless, two male specimens collected in the city of São Paulo morphologically identified as *Hg. janthinomys* (SP219, SP240; Additional file 1: Supplementary Table S1) were also clustered within this clade, reinforcing the reciprocal paraphyly of the two species, and thus indicating that the COI marker alone is insufficient to distinguish them.

**Fig. 5:**
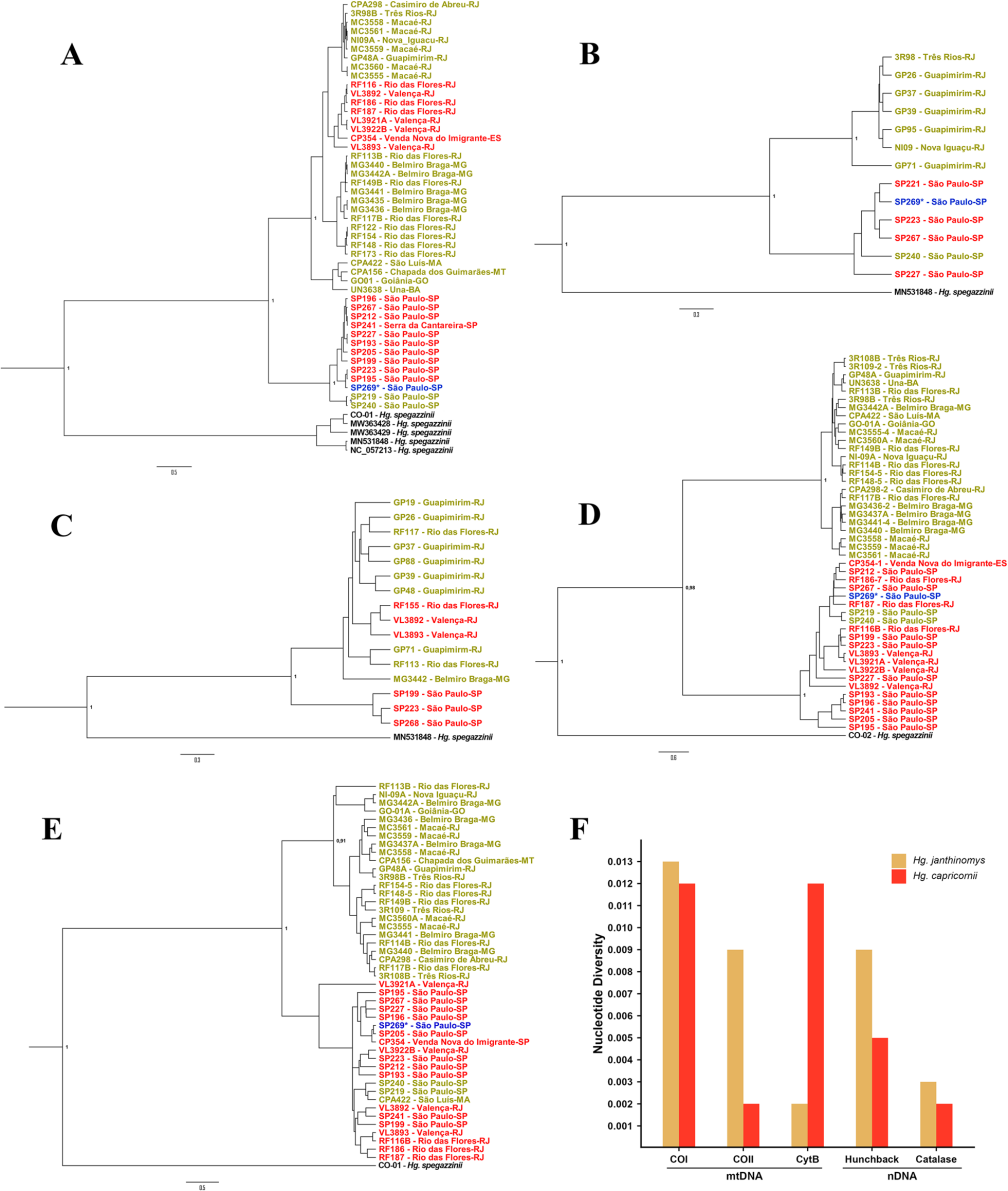
Bayesian phylogenetic reconstruction for each molecular marker and comparison of nucleotide diversities of *Hg. janthinomys* and *Hg. capricornii*. **A** COI, **B** COII, **C** CytB, **D** hunchback, and **E** catalase. Node labels indicate posterior probability values. Tip colors represent the morphological identification of specimens: dark yellow = *Hg. janthinomys*; red = *Hg. capricornii*; blue and asterisk (*) = female *Hg. janthinomys*/*Hg. capricornii*. **F** Comparison of nucleotide diversity across the five markers for *Hg. janthinomys* and *Hg. capricornii* excluding potential hybrids. Gray = all sequences of the gene

To determine whether the observed paraphyly was due to limitations of the COI marker (i.e. low-divergent fragment) or indicative of introgression or incomplete lineage sorting of the mitochondrial genome, additional analyses were conducted with two mitochondrial markers: COII and CytB. The presence of two main clades containing *Hg. janthinomys* and *Hg. capricornii* sequences was also recovered in COII and CytB phylogenetic trees, though with fewer sequences available (Fig. 5A–C). The paraphyletic relationship between *Hg. janthinomys* and *Hg. capricornii* persisted in phylogenetic reconstructions of both mitochondrial markers. One *Hg. janthinomys* sequence from the city of São Paulo (SP240) clustered with *Hg. capricornii* in COII analysis (Fig. 5B), and three *Hg. capricornii* sequences from Valença and Rio das Flores, RJ (VL3892, VL3893 and RF155), grouped with *Hg. janthinomys* in the CytB phylogenetic trees (Fig. 5C; Additional file 1: Supplementary Table S1). These specimens showed inconsistent patterns between morphological and molecular data across all mitochondrial markers, suggesting potential mitochondrial introgression.

To further explore this evolutionary relationship, *Hg. janthinomys* and *Hg. capricornii* sequences of nuclear markers (*hunchback* and *catalase*) were also analyzed (Fig. 5D, E). A specimen that could not be identified morphologically in this study (SP269) was clustered consistently within the *Hg. capricornii* clade across all phylogenetic reconstructions (with mitochondrial and nuclear markers). Interestingly, all tested specimens from Rio das Flores (RJ), Valença (RJ), and Venda Nova do Imigrante (ES) morphologically identified as *Hg. capricornii*, but previously grouped with *Hg. janthinomys* in mitochondrial tree reconstructions, clustered with *Hg. capricornii* in both nuclear analyses (RF116, RF186, RF187, RF155, VL3892, VL3893, VL3921, VL3922, and CP354). In contrast, two samples of *Hg. janthinomys* from the city of São Paulo (SP219, SP240) derived from the same female remained within the *Hg. capricornii* clade in all phylogenetic reconstructions. Moreover, one *Hg. janthinomys* from Maranhão (CPA422), where *Hg. capricornii* seems not to occur, also grouped unexpectedly within the *Hg. capricornii* clade in the phylogenetic trees based on *catalase* sequences.

Nucleotide diversity varied across the five markers analyzed, with mitochondrial and nuclear genes showing distinct patterns (Fig. 5F). Among mitochondrial markers, COI and COII exhibited the highest overall nucleotide diversity (*π* = 0.018 and 0.016, respectively), while CytB showed lower values (*π* = 0.007). When considering species separately (and excluding potential introgressed samples), *Hg. janthinomys* exhibited higher diversity than *Hg. capricornii* in COI (*π* = 0.012 versus 0.002) and COII (*π* = 0.004 versus 0.002), while in CytB, *Hg. capricornii* showed slightly higher diversity than *Hg. janthinomys* (*π* = 0.003 versus 0.002). In nuclear markers, the *hunchback* gene fragment had the highest overall nucleotide diversity (*π* = 0.021), with similar values between species (*Hg. janthinomys*: π = 0.004; *Hg. capricornii*: *π* = 0.005). In contrast, *catalase* sequences showed the lowest overall diversity (*π* = 0.002), with moderately higher values in *Hg. janthinomys* (π = 0.003) compared with *Hg. capricornii* (*π* = 0.002).

As expected, genetic distance analyses across all markers revealed consistently low divergence between *Hg. janthinomys* and *Hg. capricornii,* relative to their distances from *Hg. spegazzinii* and other *Haemagogus* species (Additional file 1: Supplementary Table S5). The interspecific divergence between COI sequences of *Hg. janthinomys* and *Hg. capricornii* was K2P = 2.4% ± 0.5, while intraspecific distances were below 1.5% (K2P = 1.2% ± 0.2 in *Hg. janthinomys* and K2P = 1.1% ± 0.2 in *Hg. capricornii*). The other mitochondrial markers and catalase also showed similar genetic distances, with interspecific divergences ranging from 2.0 to 2.6% and intraspecific divergences below 1.5%. The highest interspecific divergence between *Hg. janthinomys* and *Hg. capricornii* was observed in *hunchback* sequences (K2P = 4.2% ± 0.9), of which intraspecific divergence did not exceed 1.5% (K2P = 1.0% ± 0.2 in *Hg. janthinomys* and K2P = 0.9% ± 0.3 in *Hg. capricornii*).

#### Phylogeography

A time-calibrated phylogeny based on COI sequences using a strict molecular clock revealed that *Hg. lucifer* diverged from all other *Haemagogus* species in the Late Miocene/Early Pliocene era, approximately at 6.10 (7.46–4.86) Mya (Additional file 1: Supplementary Table S6). The clade comprising *Hg. leucocelaenus*, *Hg. equinus*, and *Hg. tropicalis* has diverged from *Hg. albomaculatus*, *Hg. mesodentatus*, *Hg. spegazzinii*, *Hg. janthinomys*, and *Hg. capricornii* in the Pliocene era, at 3.69 (4.49–2.90) Mya. The divergence between *Hg. janthinomys/capricornii* and the clade formed by *Hg. spegazzinii*, *Hg. albomaculatus*, and *Hg. mesodentatus* probably has occurred in the Late Pliocene/Early Pleistocene era, at 3.01 (3.51–2.23) Mya. This separation was similarly dated in COII (2.91 Mya, 4.65–1.54), and CytB (2.68 Mya, 4.09–1.37). Nuclear markers suggested a more ancient split between *Hg. janthinomys/capricornii* and *Hg. spegazzinii*, at 5.72 (9.73–2.59) Mya, using *hunchback* sequences, or at 4.78 (7.94–2.10) Mya, using *catalase* sequences.

The separation between *Hg. janthinomys* and *Hg. capricornii* has probably occurred in the Early/Middle Pleistocene era, at 1.20 (1.57–0.83) Mya, using COI, 1.07 (1.66–0.56) Mya with COII, and 0.89 (1.41–0.45) Mya when using CytB sequences. Nuclear markers suggested that these sister species had split earlier, at 3.24 (5.31–1.39) Mya when considering *hunchback* sequences, or 1.43 (2.23–0.79) Mya, when regarding *catalase* sequences. The split between the two *Hg. janthinomys* subclades occurred later, at 0.75 (1.00~0.54) Mya.

**Fig. 6:**
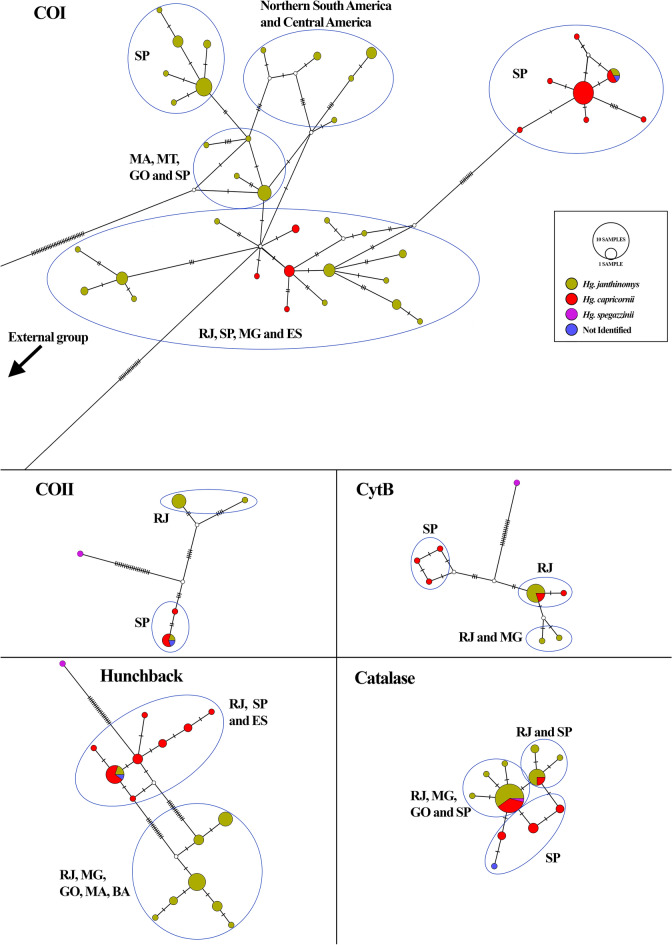
Haplotype networks inferred using the median-joining method for each molecular marker. Circle sizes are proportional to haplotype frequency. Colors represent species or clades as follows: dark yellow = *Hg. janthinomys*; red = *Hg. capricornii*; purple = *Hg. spegazzinii*; blue = specimen not morphologically identified. Haplotypes shared between species or clades are shown as pie charts, illustrating the proportion of samples from each group

Haplotype network analyses supported the separation of the main *Hg. janthinomys* and *Hg. capricornii* sequences into two groups. These groups had a geographic structuring, excepting the *catalase* network (Fig. 6). The networks based on mitochondrial markers had 9–14 mutational steps separating the major clades and showed that *Hg. capricornii* collected outside São Paulo clustered with *Hg. janthinomys*, and *Hg. janthinomys* collected in a more forested area of the Cantareira Mountain range (Guarulhos, São Paulo) grouped with *Hg. capricornii*. For *hunchback*, most haplotypes clustered separately by species, with 15–17 mutational steps between them. However, one haplotype was shared between *Hg. capricornii* samples and those *Hg. janthinomys* collected in São Paulo, previously identified as potential hybrids. In contrast, *catalase* displayed two main haplotypes, but shared between the two species. There was a minimal genetic differentiation among haplotypes, which were separated by single or few mutational steps.

Among neutrality tests, only Tajima’s D was significantly negative (−2.198; *P* = 0.03) in COI for the clade dominated by *Hg. capricornii*. Most of the other neutrality tests statistics, including Tajima’s D, Fu’s F, and Fu and Li’s D and F, were also negative across all markers, although not statistically significant (Additional file 1: Supplementary Table S7). These results suggested possible demographic expansion of *Hg. capricornii*, but with weak statistical support.

**Fig. 7:**
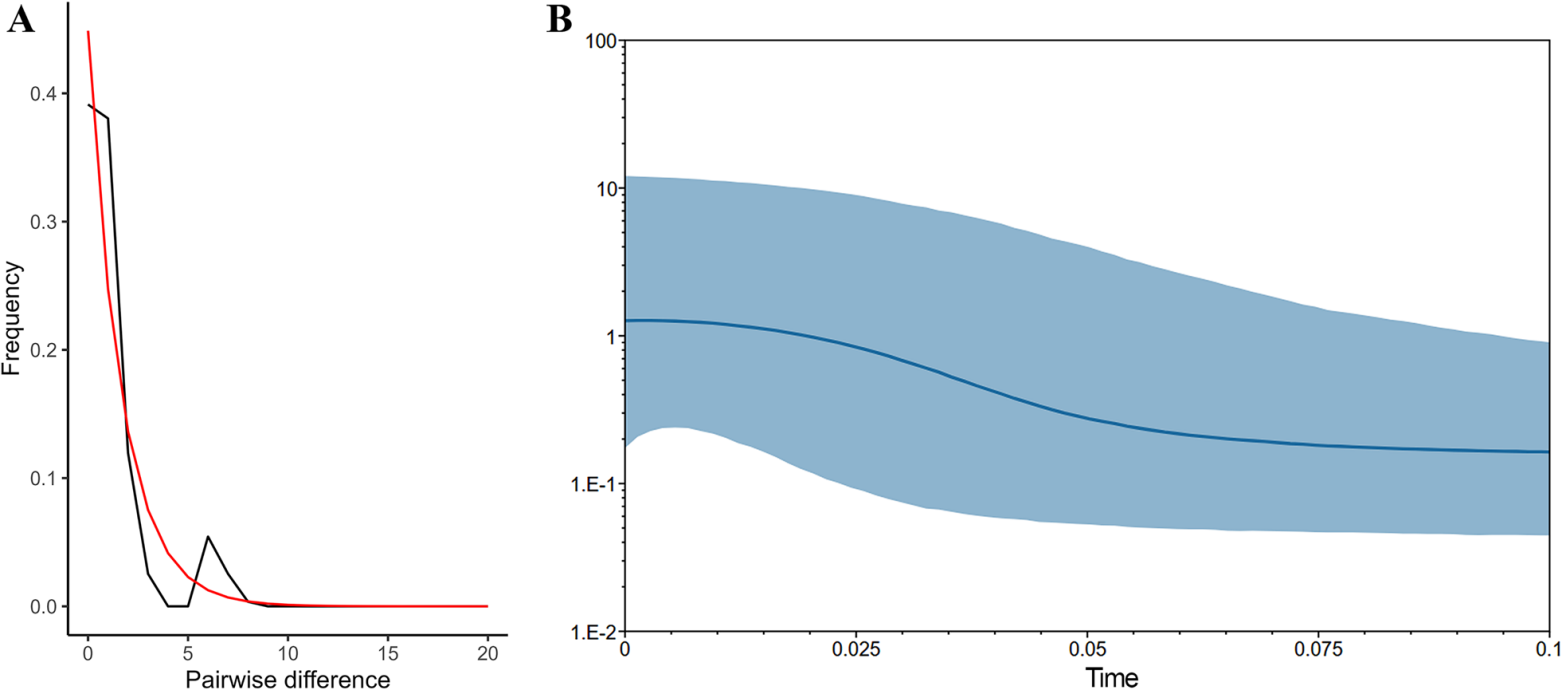
Population analyses based on *Hg. capricornii* mt-COI sequences. **A** Mismatch distribution. The black line represents the observed distribution of pairwise nucleotide differences, and the red line indicates the expected distribution under a model of sudden demographic expansion. **B** Bayesian Skyline Plot (BSP). The blue line shows changes in effective population size over time, while the shaded area represents the 95% highest posterior density interval. The x-axis represents time in millions of years before present, and the y-axis corresponds to relative effective population size

Mismatch distribution analysis supported a signal of recent expansion in the *Hg. capricornii* COI clade, with a unimodal distribution and a low raggedness index (*r* = 0.0471; Fig. 7A). Bayesian Skyline Plot (BSP) analysis further indicated a population expansion in *Hg. capricornii*, from which it is possible to estimate that its population triplicated between 60 and 20 thousand years ago (Fig. 7B). In contrast, mismatch distributions and BSPs of *Hg. janthinomys*, whether analyzed as a single group or divided into the two subclades observed in the phylogenetic tree, did not exhibit features indicative of demographic expansion (Additional file 2: Supplementary Fig. S3).

## Discussion

*Haemagogus janthinomys* and *Hg. capricornii* have long been subject to taxonomic controversy, primarily owing to the limited number of morphological characters distinguishing them. *Haemagogus capricornii* was first described by Lutz in 1904 [6] based on female specimens only, while *Hg. janthinomys* was described in 1921 by Dyar [5], who later in 1923 synonymized *Hg. capricornii* with *Hg. equinus* based solely on female samples—reviewed in Arnell [10]. Antunes [42] revalidated *Hg. capricornii* and made *Hg. janthinomys* its junior synonym, but Cerqueira [43] synonymized *Hg. janthinomys* with *Hg. spegazzinii*, while Cerqueira and Lane [44] confirmed the validity of *Hg. capricornii*, including female and male specimens from the type locality (Horto Florestal da Cantareira, São Paulo, Brazil) in a redescription, and also elected a female neotype.

Once the male genitalia were described for *Hg. janthinomys* (as *Hg. spegazzinii*) and *Hg. capricornii*, Martinez et al. [45] found variations in the apical process of the aedeagus, with long, intermediate, and short tips, and thus placed these species into three subspecies: *Hg. capricornii falco* [46], *Hg. c. janthinomys*, and *Hg. c. petrocchiae* [45]. Arnell [11], however, did not find a geographical pattern in those morphological features, but two differences in the male genitalia were consistently distinct between *Hg. janthinomys* and *Hg. capricornii*: the apex of the paraproct with a hooklike process and spicules on the upper distal mesal area of the aedeagus in *Hg. janthinomys*, but not in *Hg. capricornii,* as previously described and illustrated by Cerqueira [43]. These differences have been used since then to differentiate the two species.

The taxonomical differentiation of the two species is of great epidemiological relevance, as *Hg. janthinomys* is considered a primary vector of YFV in sylvatic environments, and has an extensive geographical distribution, from Central America to northern Argentina [47]. On the other hand, *Hg. capricornii* occupies a restricted region in south-southeastern Brazil, within the Atlantic Forest biome, and since its females are cryptic in relation to *Hg. janthinomys*, its role in YFV transmission has been eclipsed, and natural infection rates during YF outbreaks are unknown or uncertain [7, 10, 11]. Moreover, because the adult males of both species are too difficult to be collected in the field, modern techniques such as DNA-based analyses are challenging to integrate with morphology.

The first molecular study (and the only known so far) involving an evolutionary analysis of the two species was recently conducted by Telles-de-Deus et al. [18]. The authors analyzed 39 specimens, of which only two male specimens were morphologically identified as *Hg. capricornii*, while specific identification of the remaining 37 individuals was not confirmed. All specimens were collected in nine municipalities restricted to SP and had the “universal” COI DNA barcoding fragment [21] sequenced. Their results revealed two main genetic clades, which they attributed to belonging to *Hg. janthinomys* and *Hg. capricornii*, and thus concluded that COI sequences would be effective in differentiating these *Haemagogus* species. In the present study, we carried out a more comprehensive phylogenetic analyses by sequencing two mitochondrial (COII, and CytB) and two nuclear (*hunchbach* and *catalase*) gene fragments besides COI of 79 male specimens whose species identity was morphologically confirmed by genitalia examination (56 *Hg. janthinomys* and 23 *Hg. capricornii*), and two female siblings of morphologically identified males as *Hg. janthinomys*. The specimens were collected in eight Brazilian states (BA, ES, MA, MG, MT, RJ, RO, and SP). In two municipalities (Rio das Flores, RJ, and São Paulo, SP), both species were found in sympatry, and were collected at the same site within a 2-hour interval on the same day.

In this study, phylogenetic and network analyses displayed two main clades with a geographic structure, as previously described Telles-de-Deus et al. [18], but with patterns of reciprocal paraphyly between the two species. Moreover, it was observed as a disagreement between mitochondrial and nuclear tree reconstructions, which are suggestive of ancient split followed by possible recent hybridization. The phylogenetic reconstructions and networks based on mitochondrial markers evidenced a clade that consistently grouped all *Hg. janthinomys* (but those from the Cantareira Mountain range in São Paulo) with all sequences of *Hg. capricornii* samples collected outside São Paulo (Valença and Rio das Flores, RJ, and Venda Nova do Imigrante, ES), while the other clade clustered the two *Hg. janthinomys* from São Paulo with the sympatric *Hg. capricornii* counterparts. Regarding the phylogenetic reconstructions based on nuclear markers, we observed that *Hg. capricornii* was monophyletic (i.e. all sequences of *Hg. capricornii* samples clustered together irrespective of their geographical origin), but *Hg. janthinomys* was not. The paraphyletic assemblage was evidenced in mitochondrial and nuclear markers by the two *Hg. janthinomys* collected in São Paulo that consistently clustered with *Hg. capricornii*.

Mitochondrial markers are widely used and are still vital for insect evolutionary studies, owing to several reasons, including the large sequence datasets that publicly available, its applicability across most animal phyla for species identification, adequate mutation rate to access potential differences between closely related species, and the ease of sequencing [48–50]. Moreover, since it is maternally inherited, it completes the process of lineage sorting faster than nuclear markers, and thus reflects better recent divergences. Indeed, a review on cryptic speciation revealed that the COI gene was used in 83% of the analyzed studies [51]. On the other hand, however, the uniparental inheritance also makes them more prone to changes in the phylogenetic signal than nuclear markers due to introgression, which require extensive backcrossing to be similarly affected [50]. Therefore, mitonuclear discordances can be observed if ancestral populations experienced periods of geographic isolation, followed by subsequent secondary contact [48, 52].

The phylogenetic reconstructions based on the *hunchback* gene evidenced a quasi-reciprocal monophyly of *Hg. capricornii* and *Hg. janthinomys*, and probably reflects a scenario of ancient divergence between the two species. Although the estimated median divergence time varied among the molecular markers, the confidence intervals across all datasets suggest that this separation likely occurred during the Early Pleistocene era, approximately at 1.5 Mya. This period was marked for diversification in neotropical taxa, with climatic oscillations and habitat shifts [53], and the Atlantic Forest served as a historical refuge for species during glacial cycles [16, 54, 55]. Given the wide geographical distribution of *Hg. janthinomys* and the limited range of *Hg. capricornii*, we hypothesize that their ancestral population was probably more generalist, occupying a wide area in Central and South America, and part of this population may have diverged in parapatry or peripatry probably in São Paulo. In this case, *Hg. capricornii* would be more associated with the Atlantic Forest and the semi-deciduous forests of plateau areas [56], and these highland regions may have played a role as forest glacial refugia, maintaining during this period their ancestral populations isolated from *Hg. janthinomys*. Indeed, reliable geographic records of the distribution of these two species, based exclusively on examination of male genitalia, as made herein, confirm that *Hg. capricornii* tends to be much more frequent in the humid Atlantic Forest at altitudes from 400 to 1000 m, on mountain slopes and valleys. In contrast, *Hg. janthinomys* spans a wide range of environments, biomes, and phytogeographic regions from the southern half of Central America to northern Argentina, becoming gradually less frequent as it approaches the Paulista Plateau, occurring in both lowlands and highlands, suggesting that *Hg. janthinomys* has much greater ecological plasticity, thriving in a wide range of environmental conditions [11, 47, 57].

The wide distribution of *Hg. janthinomys* results in highly diverse populations, although pairwise divergences were consistent with intraspecific variation (K2P_COI_ < 1.5%) in Culicidae mosquitoes [58]. The two subclades observed in COI phylogenetic reconstructions within *Hg. janthinomys* have diversified around 0.75 Mya, and possibly reflect a complex evolutionary history involving population subdivision and geographic isolation. Their ancestral populations, however, appear to have remained highly stable over the past 0.2 Mya in terms of geographical occurrence and population size, which was evidenced by nonsignificant results of neutrality tests and constant effective population sizes in BSPs (Additional file 1: Supplementary Table S7; Additional file 2: Supplementary Fig. S3). The two observed subclades were comprised by samples collected throughout Central America and South America, while the other subclade was geographically more restricted, comprising exclusively mosquitoes from the Atlantic Forest biome of southeastern Brazil.

In contrast, *Hg. capricornii* sequences were less diverse, clustering into a single group, but showing evidence of recent demographic expansion around 60–20 thousand years ago, when the mosquito population tripled (Fig. 6). This period was characterized by cooler temperatures (5 °C lower than present), and the region was dominated by semideciduous and evergreen forests [55]. The mitonuclear incompatibilities observed in *Hg. capricornii* samples from outside São Paulo may be explained by this sudden expansion, which likely led to a secondary contact with *Hg. janthinomys* in areas outside São Paulo, at the Paraiba valley (Rio das Flores and Valença, Rio de Janeiro) and Espírito Santo, possibly already occupied by the latter species.

The presence of two morphologically identified *Hg. janthinomys* samples from São Paulo in the *Hg. capricornii* clade in all phylogenetic reconstructions could be explained by the fact that the distribution of *Hg. janthinomys* marginally overlaps with that of *Hg. capricornii* and when in contact, they could hybridize. In this case, owing to the larger number of *Hg. capricornii* in the city of São Paulo, successive breeding over many generations would have incorporated more gene pools of *Hg. capricornii* on these samples morphologically identified as *Hg. janthinomys*. We could not rule out the possibility of this genetic similarity reflecting the retention of ancestral polymorphism, maybe resulting from a sympatric speciation event. However, sympatric speciation remains one of the most debated modes of speciation, as strong interspecific gene flow tends to hinder population divergence. Several cases previously interpreted as examples of sympatric speciation have later been shown to involve a past allopatry when molecular divergence accumulated prior to secondary contact and sympatry (reviewed in Pavan et al. [59]). Additional evidence from multiple independent nuclear loci, denser sampling, and explicit phylogenomic modeling would be required before completely excluding incomplete lineage sorting (ILS) as the cause of the observed mitochondrial paraphyly.

Incomplete reproductive isolation and ongoing gene flow among closely related species have been widely documented in insects (reviewed in Zheng [60] and San Jose et al. [61]). In animals, the introgression of mitochondrial DNA is more frequent than nuclear DNA, and this phenomenon is often asymmetric between populations [50, 62]. In a longitudinal analysis between *Aedes mariae* and *Ae. zammitii*, it was possible to observe that mitochondrial introgression occurred earlier and more frequently in the introduced species than in the resident species [62]. Although the malaria vectors *Anopheles gambiae* Giles, 1902, *An. coluzzii* Coetzee and Wilkerson, 2013 and *An. fontenillei* Barrón et al. 2019 (Diptera: Culicidae) are considered valid species, introgressed genomic regions have been also identified, including genes associated with detoxification, desiccation tolerance, and olfactory perception, traits that can influence their vectorial capacity [63]. Moreover, the *Culex pipiens* L. complex that includes *Cx. pipiens* Linnaeus, 1758 and *Cx. quinquefasciatus* Say, 1823 occurs in North America, South America, Africa, and Asia. These species are vectors of the West Nile Virus (WNV) and are capable of hybridizing in regions where their populations overlap. Ciota et al. [64] found that hybrids carrying the genomic signature of *Cx. quinquefasciatus* showed increased susceptibility to WNV infection, which means that both *Cx. quinquefasciatus* and hybrids were more susceptible than *Cx. pipiens*. In the case of *Haemagogus*, further studies integrating molecular taxonomy, arbovirus infection rates, and vector competence assays are needed to determine whether introgressed individuals differ in their epidemiological roles and how hybridization may influence YFV and other arboviruses transmission dynamics.

The presence of one sample of *Hg. janthinomys* from São Luís, Maranhão, clustering together with *Hg. capricornii* in the phylogenic tree based on *catalase* sequences seems to have resulted from ILS. Among the five markers analyzed, *catalase* had the overall lowest genetic diversity (four times lower than that of CytB, which was the second least diverse marker), and its network with shared haplotypes did not reveal a clear separation between the two species (Figs. 5F and 6). Additionally, this region is located at least 1600 km from the nearest known area of *Hg. capricornii* occurrence.

In our COI phylogeny, sequences from Telles-de-Deus et al. [18] were distributed across multiple clades: some grouped with sequences from specimens we morphologically confirmed to be *Hg. janthinomys*, while others clustered with *Hg. capricornii*. This suggests that their dataset may include misidentified *Hg. capricornii* or hybrids labeled as *Hg. janthinomys*, potentially influencing their estimates of diversity and population structure. Given the evidence of a secondary contact zone in SP where both species are found in sympatry and hybridization likely occurs, it is possible that additional introgressed individuals are present in their dataset.

Mitochondrial genes, although widely used in insect phylogenetics, typically reflect maternal lineages and evolutionary events that occurred within the past tens to hundreds of thousands of years. Therefore, they may not fully capture more recent or ongoing gene flow between species. In this sense, the nuclear genes analyzed represent only a small fraction of the genome and may not offer a comprehensive view of the extent or direction of introgression. To more closely investigate the presence of gene flow, future studies should incorporate fast-evolving markers such as microsatellites, or genome-wide sequencing. These techniques would allow a finer resolution of hybridization patterns and help clarify if the observed mitonuclear discordance reflects historical introgression or active genetic exchange between species.

## Conclusions

Overall, our results confirm the validity of *Hg. janthinomys* and *Hg. capricornii* as closely related species, but with distinct evolutionary lineages. We analyzed a comprehensive molecular dataset in which nearly all sequences were reliably associated with morphologically identified specimens, and, contrary to what Telles-de-Deus et al. [18] suggested, DNA barcoding based on COI sequences alone could not differentiate *Hg. janthinomys* from *Hg. capricornii*. Even with the inclusion of two additional mitochondrial and two nuclear markers, we did not observe a clear reciprocal monophyly. The phylogenetic structure, morphological differentiation in male genitalia, and demographic patterns support the scenario of a Pleistocene divergence of the species, followed by spatial overlap and episodes of gene flow. The evidence of reciprocal paraphyly and mitonuclear discordance, especially in southeastern Brazil, suggests that hybridization may still be shaping the genetic background of these mosquitoes. This emphasizes the complexity of delimiting cryptic species and the need to integrate evidence in taxonomic and evolutionary studies to improve species identification for surveillance and public health interventions, particularly in areas of sympatry and potential hybridization. Future work using fast-evolving markers or genome-wide comparisons are needed to define possible contemporary gene flow between the two species. This information coupled with mosquito infection rates should help clarify the epidemiological scenario in southeastern Brazil involving potential hybrid zones.

## Supplementary Information

Additional file 1.

Additional file 2.

## Data Availability

All data generated and analyzed during this study are included in this published article and its supplementary information files. DNA sequences were deposited in the GenBank (https://www.ncbi.nlm.nih.gov/genbank/) under the Submission IDs 3012149 and 3013925.

## Abbreviations

BSP: Bayesian Skyline Plot
Cat: Catalase gene
COI: Cytochrome c oxidase subunit I gene
COII: Cytochrome c oxidase subunit II gene
CytB: Cytochrome B gene
hb: Hunchback gene
Mya: Million years ago
PP: Posterior probability
UFBoot: Ultrafast bootstrap
YF: Yellow fever
YFV: Yellow fever virus

## References

[1] Monath TP, Vasconcelos PFC. Yellow fever. J Clin Virol. 2015;64:160–73. 10.1016/j.jcv.2014.08.030. 25453327 10.1016/j.jcv.2014.08.030

[2] Brasil, Saúde M da. Monitoramento da febre amarela – 2020/2021. Boletim Epidemiológico Arboviroses. 2021.

[3] Possas C, Lourenço-de-Oliveira R, Tauil PL, Pinheiro FD, Pissinatti A, Cunha RV, Freire M, Martins RM, Homma A. Yellow fever outbreak in Brazil: the puzzle of rapid viral spread and challenges for immunisation. Mem Inst Oswaldo Cruz. 2018;113. 10.1590/0074-02760180278 10.1590/0074-02760180278 PMC6135548 30427974

[4] Dyar HG, Shannon RC. The subfamilies, tribes, and genera of American Culicidae. Source: Journal of the Washington Academy of Sciences. 1924.

[5] Dyar HG. The genus *Haemagogus* Williston. (Diptera, Culicidae.). Insecutor inscitiae menstruus. 1921;IX:101–14.

[6] Adolfo Lutz. Chave para a determinação das espécies de Euculicidae encontradas no Brasil (não incluindo a subfamília Culicinae). In Bourroul C. Mosquitos do Brasil. Faculdade de Saúde Bahia; 1904.

[7] Abreu FV, Ribeiro IP, Ferreira-de-Brito A, Santos AA, Miranda RM, Bonelly ID, et al. *Haemagogus leucocelaenus* and *Haemagogus janthinomys* are the primary vectors in the major yellow fever outbreak in Brazil, 2016–2018. Emerg Microbes Infect. 2019;8:218–31. 10.1080/22221751.2019.1568180. 30866775 10.1080/22221751.2019.1568180 PMC6455131

[8] Cunha MS, da Costa AC, de Azevedo Fernandes NC, Guerra JM, Dos Santos FC, Nogueira JS, et al. Epizootics due to yellow fever virus in São Paulo State, Brazil: viral dissemination to new areas (2016–2017). Sci Rep. 2019;9:5474. 10.1038/s41598-019-41950-3. 30940867 10.1038/s41598-019-41950-3 PMC6445104

[9] Pinheiro GG, Rocha MN, de Oliveira MA, Moreira LA, Andrade Filho JD. Detection of yellow fever virus in sylvatic mosquitoes during disease outbreaks of 2017–2018 in Minas Gerais State. Brazil Insects. 2019;10:136. 10.3390/insects10050136. 31083286 10.3390/insects10050136 PMC6572267

[10] Stanzani LM, Motta MD, Erbisti RS, Abreu FV, Nascimento-Pereira AC, Ferreira-de-Brito A, et al. Back to where it was first described: vectors of sylvatic yellow fever transmission in the 2017 outbreak in Espírito Santo. Brazil Viruses. 2022;14:2805. 10.3390/v14122805. 36560809 10.3390/v14122805 PMC9785321

[11] Arnell JH. Mosquito studies (Diptera, Culicidae) XXXII. A revision of the genus *Haemagogus*. Contributions of the American Entomological Institute 1973;10. http://mosquito-taxonomic-inventory.info/mosquito-studies-diptera-culicidae-xxxii-revision-genus-ltemgthaemagogusltemgt. Accessed 25 Sep 2025.

[12] Lutz A. Catalogo dos culicideos brasileiros e sul-americanos. Synopse e systhematisação dos mosquitos do Brasil. 1904;7:1–16.

[13] Reidenbach KR, Cook S, Bertone MA, Harbach RE, Wiegmann BM, Besansky NJ. Phylogenetic analysis and temporal diversification of mosquitoes (Diptera: Culicidae) based on nuclear genes and morphology. BMC Evol Biol. 2009;9:298. 10.1186/1471-2148-9-298. 20028549 10.1186/1471-2148-9-298 PMC2805638

[14] Beebe NW. DNA barcoding mosquitoes: advice for potential prospectors. Parasitology. 2018;145:622–33. 10.1017/S0031182018000343. 29564995 10.1017/S0031182018000343

[15] Lemos PD, Monteiro HA, Castro FC, Lima CP, Silva DE, Vasconcelos JM, et al. Characterization of mitochondrial genome of *Haemagogus janthinomys* (Diptera: Culicidae). Mitochondrial DNA Part A. 2017;28:50–1. 10.3109/19401736.2015.1110793. 10.3109/19401736.2015.1110793 26709451

[16] da Silva AF, Machado LC, de Paula MB, da Silva Pessoa Vieira CJ, de Morais Bronzoni RV, de Melo Santos MA, Wallau GL. Culicidae evolutionary history focusing on the Culicinae subfamily based on mitochondrial phylogenomics. Sci Rep. 2020;10:1–14. 10.1038/s41598-020-74883-3 10.1038/s41598-020-74883-3 PMC7606482 33139764

[17] Oliveira TMP, Saraiva JF, da Silva H, Sallum MAM. Molecular Identification of mosquitoes (Diptera: Culicidae) using COI barcode and D2 expansion of 28S gene. DNA. 2024;4:507–18. 10.3390/dna4040034.

[18] Telles-de-Deus J, de Oliveira GL, Rocha EC, Helfstein VC, Reginato SL, Mucci LF, et al. COI DNA barcoding to differentiate *Haemagogus janthinomys* and *Haemagogus capricornii* (Diptera: Culicidae) mosquitoes. Acta Trop. 2024;259:107377. 10.1016/j.actatropica.2024.107377. 39245155 10.1016/j.actatropica.2024.107377

[19] Couto-Lima D, Andreazzi CS, Leite PJ, Bersot MIL, Alencar J, Lourenço-de-Oliveira R. Seasonal population dynamics of the primary Yellow Fever vector *Haemagogus leucocelaenus* (Dyar & Shannon) (Diptera: Culicidae) is mainly influenced by temperature in the Atlantic Forest, southeast Brazil. Mem Inst Oswaldo Cruz. 2020;115:1–13. 10.1590/0074-02760200218. 10.1590/0074-02760200218 PMC7370926 32696917

[20] Ribeiro PS, Galvao C, Talaga S, Carinci R, Pavan MG, Lourenco-de-Oliveira R, Motta MD. Redescription and placement of *Wyeomyia rorotai* Senevet, Chabelard & Abonnenc (Diptera: Culicidae) in the subgenus *Decamyia* based on morphological and molecular analyses. Zootaxa. 2020;4830:291–309. 10.11646/zootaxa.4830.2.4 10.11646/zootaxa.4830.2.4 33056153

[21] Hebert PDN, Cywinska A, Ball SL, DeWaard JR. Biological identifications through DNA barcodes. Proc R Soc Lond B Biol Sci. 2003;270:313–21. 10.1098/rspb.2002.2218. 10.1098/rspb.2002.2218 PMC1691236 12614582

[22] Folmer O, Black M, Hoeh W, Lutz R, Vrijenhoek R. DNA primers for amplification of mitochondrial cytochrome c oxidase subunit I from diverse metazoan invertebrates. Mol Mar Biol Biotechnol. 1994;3:294–9. 10.1016/j.ijbiomac.2019.12.135. 7881515

[23] Kumar S, Stecher G, Li M, Knyaz C, Tamura K. MEGA X: molecular evolutionary genetics analysis across computing platforms. Mol Biol Evol. 2018;35:1547–9. 10.1093/molbev/msy096. 29722887 10.1093/molbev/msy096 PMC5967553

[24] Untergasser A, Nijveen H, Rao X, Bisseling T, Geurts R, Leunissen JAM. Primer3Plus, an enhanced web interface to Primer3. Nucleic Acids Res. 2007;35:W71–4. 10.1093/nar/gkm306. 17485472 10.1093/nar/gkm306 PMC1933133

[25] Katoh K, Standley DM. MAFFT multiple sequence alignment software version 7: improvements in performance and usability. Mol Biol Evol. 2013;30:772–80. 10.1093/molbev/mst010. 23329690 10.1093/molbev/mst010 PMC3603318

[26] RStudio Team. RStudio: integrated development for R. RStudio [Internet]. PBC, Boston, MA. 2023. http://www.rstudio.com/

[27] Paradis E, Schliep K. ape 5.0: an environment for modern phylogenetics and evolutionary analyses in R. Schwartz R, editor. Bioinformatics. 2019;35:526–8. 10.1093/bioinformatics/bty633 10.1093/bioinformatics/bty633 30016406

[28] Paradis E. pegas: an R package for population genetics with an integrated–modular approach. Bioinformatics. 2010;26:419–20. 10.1093/bioinformatics/btp696. 20080509 10.1093/bioinformatics/btp696

[29] Wickham H. ggplot2: elegant graphics for data analysis. 2nd ed. Springer Cham; 2016. 10.1007/978-3-319-24277-4

[30] Minh BQ, Schmidt HA, Chernomor O, Schrempf D, Woodhams MD, Von Haeseler A, et al. IQ-TREE 2: New models and efficient methods for phylogenetic inference in the genomic era. Teeling E, editor. Mol Biol Evol. 2020;37:1530–4. 10.1093/molbev/msaa015. 32011700 10.1093/molbev/msaa015 PMC7182206

[31] Bouckaert R, Vaughan TG, Barido-Sottani J, Duchêne S, Fourment M, Gavryushkina A, et al. BEAST 2.5: an advanced software platform for Bayesian evolutionary analysis. PLoS Comput Biol. 2019. 10.1371/journal.pcbi.1006650. 30958812 10.1371/journal.pcbi.1006650 PMC6472827

[32] Guindon S, Dufayard J-FF, Lefort V, Anisimova M, Hordijk W, Gascuel O. New algorithms and methods to estimate Maximum-Likelihood phylogenies: assessing the performance of PhyML 3.0. Syst Biol. 2010;59:307–21. 10.1093/sysbio/syq010 10.1093/sysbio/syq010 20525638

[33] Bouckaert RR, Drummond AJ. bModelTest: Bayesian phylogenetic site model averaging and model comparison. BMC Evol Biol. 2017;17:1–11. 10.1186/s12862-017-0890-6. 28166715 10.1186/s12862-017-0890-6 PMC5294809

[34] Heled J, Drummond AJ. Bayesian inference of species trees from multilocus data. Mol Biol Evol. 2010;27:570–80. 10.1093/molbev/msp274. 19906793 10.1093/molbev/msp274 PMC2822290

[35] Rambaut A, Drummond AJ, Xie D, Baele G, Suchard MA. Posterior summarization in bayesian phylogenetics using Tracer 1.7. Susko E, editor. Syst Biol. 2018;67:901–4. 10.1093/sysbio/syy032 10.1093/sysbio/syy032 PMC6101584 29718447

[36] Tiley GP, Poelstra JW, dos Reis M, Yang Z, Yoder AD. Molecular clocks without rocks: new solutions for old problems. Trends Genet. 2020;36:845–56. 10.1016/j.tig.2020.06.002. 32709458 10.1016/j.tig.2020.06.002

[37] Papadopoulou A, Anastasiou I, Vogler AP. Revisiting the insect mitochondrial molecular clock: the mid-aegean trench calibration. Mol Biol Evol. 2010;27:1659–72. 10.1093/molbev/msq051. 20167609 10.1093/molbev/msq051

[38] Kimura M. A simple method for estimating evolutionary rates of base substitutions through comparative studies of nucleotide sequences. J Mol Evol. 1980;16:111–20. 10.1007/BF01731581. 7463489 10.1007/BF01731581

[39] Kassambara A. ggpubr R Package: ggplot2-Based Publication Ready Plots [Internet]. 2023. https://www.sthda.com/eng-%0Alish/articles/24-ggpubr-publication-ready-plots/%0A

[40] Wilke C. Introduction to ggridges [Internet]. 2024. https://wilkelab.org/ggridges/articles/introduction.html/

[41] Rozas J, Ferrer-Mata A, Sánchez-DelBarrio JC, Guirao-Rico S, Librado P, Ramos-Onsins SE, et al. DnaSP 6: DNA sequence polymorphism analysis of large data sets. Mol Biol Evol. 2017;34:3299–302. 10.1093/molbev/msx248. 29029172 10.1093/molbev/msx248

[42] Antunes P. Antunes PCA. Nota sobre o genero *Haemagogus* Williston (Diptera, Culicidae). Rev Paul Med. 1939;14.

[43] Cerqueira NL. Algumas espécies novas da Bolívia, e referência a três espécies *Haemagogus*. Mem Inst Oswaldo Cruz. 1943;39:1–14. 10.1590/S0074-02761943000400001.

[44] Cerqueira NL, Lane J. Note on *Haemagogus capricornii* Lutz, 1904 (Díptera, Culicidae). Proc Entomol Soc Wash. 1947;47:279–88.

[45] Martínez A, Carcavallo RU, Prosen AF. El género *Haemagogus* Willinston, 1896, en la Argentina.(Diptera: Culicidae). Anales del Instituto de Medicina Regional. 1961;5:63–86.

[46] Kumm HW, Osorno-mesa E, Boshell-manrique J. Studies on mosquitoes of the genus *Haemagogus* in Colombia (Diptera, Culicidae). Am J Epidemiol. 1946;43:13–28. 10.1093/OXFORDJOURNALS.AJE.A119048. 10.1093/oxfordjournals.aje.a119048 21011557

[47] Celone M, Pecor DB, Potter A, Richardson A, Dunford J, Pollett S. An ecological niche model to predict the geographic distribution of *Haemagogus janthinomys*, Dyar, 1921 a yellow fever and Mayaro virus vector, in South America. PLoS Negl Trop Dis 2022;16. 10.1371/journal.pntd.0010564 PMC9299311 35802748

[48] Ballard JWO, Whitlock MC. The incomplete natural history of mitochondria. Mol Ecol. 2004;13:729–44. 10.1046/j.1365-294X.2003.02063.x. 15012752 10.1046/j.1365-294x.2003.02063.x

[49] Cameron SL. Insect mitochondrial genomics: a decade of progress. Annu Rev Entomol. 2025;70:83–101. 10.1146/annurev-ento-013024-015553. 39259965 10.1146/annurev-ento-013024-015553

[50] Toews DPL, Brelsford A. The biogeography of mitochondrial and nuclear discordance in animals. Mol Ecol. 2012;21:3907–30. 10.1111/j.1365-294X.2012.05664.x. 22738314 10.1111/j.1365-294X.2012.05664.x

[51] Fišer C, Robinson CT, Malard F. Cryptic species as a window into the paradigm shift of the species concept. Mol Ecol. 2018;27:613–35. 10.1111/mec.14486. 29334414 10.1111/mec.14486

[52] Després L. One, two or more species? Mitonuclear discordance and species delimitation. Mol Ecol. 2019;28:3845–7. 10.1111/mec.15211. 31515862 10.1111/mec.15211

[53] Bennett KL, Shija F, Linton YM, Misinzo G, Kaddumukasa M, Djouaka R, et al. Historical environmental change in Africa drives divergence and admixture of *Aedes aegypti* mosquitoes: a precursor to successful worldwide colonization? Mol Ecol. 2016;25:4337–54. 10.1111/mec.13762. 27439067 10.1111/mec.13762

[54] Haffer J. Speciation in Amazonian forest birds. Science. 1979;1969:131–7. 10.1126/science.165.3889.131. 10.1126/science.165.3889.131 17834730

[55] Francisquini MI, Lorente FL, Pessenda LC, Junior AA, Mayle FE, Cohen MC, et al. Cold and humid Atlantic Rainforest during the last glacial maximum, northern Espírito Santo state, southeastern Brazil. Quat Sci Rev. 2020. 10.1016/j.quascirev.2020.106489.

[56] Forattini OP. Culicidologia Médica: Identificação, Biologia, Epidemiologia. 2nd ed. São Paulo: Editora da Universidade de São Paulo; 2002.

[57] Alencar J, dos Santos Silva J, Maués Serra-Freire N, Érico Guimarães A. Dispersion and ecological plasticity patterns of *Haemagogus capricornii* and *H. janthinomys* (Diptera: Culicidae) Populations in Different Regions of Brazil. Entomol News 2009;120:53–60. 10.3157/021.120.0111

[58] Ribeiro PS, Pavan MG, da Silva MB, Galvão C, Lourenço-de-Oliveira R, Motta MA. A new species of *Wyeomyia* (Diptera: Culicidae) from *Heliconia* flower bracts in northern South America. Zootaxa. 2021;4999:534–52. 10.11646/zootaxa.4999.6.2 10.11646/zootaxa.4999.6.2 34811327

[59] Pavan MG, Lazoski C, Monteiro FA. Speciation Processes in Triatominae. Springer, Cham; 2021, 39–64. 10.1007/978-3-030-64548-9_3

[60] Zheng XL. Unveiling mosquito cryptic species and their reproductive isolation. Insect Mol Biol. 2020;29:499–510. 10.1111/imb.12666. 32741005 10.1111/imb.12666 PMC7754467

[61] San Jose M, Doorenweerd C, Rubinoff D. Genomics reveals widespread hybridization across insects with ramifications for species boundaries and invasive species. Curr Opin Insect Sci. 2023;58:101052. 10.1016/j.cois.2023.101052. 37150509 10.1016/j.cois.2023.101052

[62] Mastrantonio V, Porretta D, Urbanelli S, Crasta G, Nascetti G. Dynamics of mtDNA introgression during species range expansion: insights from an experimental longitudinal study. Sci Rep. 2016;6:30355. 10.1038/srep30355. 27460445 10.1038/srep30355 PMC4962091

[63] Barrón MG, Paupy C, Rahola N, Akone-Ella O, Ngangue MF, Wilson-Bahun TA, et al. A new species in the major malaria vector complex sheds light on reticulated species evolution. Sci Rep. 2019;9:14753. 10.1038/s41598-019-49065-5. 31611571 10.1038/s41598-019-49065-5 PMC6791875

[64] Ciota AT, Chin PA, Kramer LD. The effect of hybridization of *Culex pipiens* complex mosquitoes on transmission of West Nile virus. Parasit Vectors. 2013;6:1–4. 10.1186/1756-3305-6-305/FIGURES/1. 24499581 10.1186/1756-3305-6-305 PMC4029739

