# Supplementary material for: Mitochondrial introgression hampers the DNA barcoding of cryptic yellow fever vectors Haemagogus capricornii Lutz and Hg. janthinomys in the Atlantic Forest, Brazil
Source: Parasit Vectors. 2026 May 11;19:211. doi: 10.1186/s13071-026-07343-y (PMC13169768; doi:10.1186/s13071-026-07343-y)
Supplement: Supplementary file 2 — Additional file 2. [file 13071_2026_7343_MOESM2_ESM.docx]

**Mitochondrial introgression hampers the DNA Barcoding of cryptic yellow fever vectors *Haemagogus capricornii* Lutz and *Hg. janthinomys* in the Atlantic Forest, Brazil**

**Filipe Vieira Santos de Abreu^1,2,#^; Lucas Bonato Mosmann^1,#^; Carolina Boucinha Martins^1^, Alexandre da Silva Xavier^1^, Igor Mello da Rocha Corpas Maciel^1^, Agostinho Cardoso Nascimento-Pereira^1,3,4^; Paulino Siqueira Ribeiro^1^; Jeronimo Alencar^3^; Rosa Maria Tubaki^5^; Monique Albuquerque Motta^1^; Ricardo Lourenço-de-Oliveira^1*^; Márcio Galvão Pavan^1*^**

^1^ Laboratório de Mosquitos Transmissores de Hematozoários, Instituto Oswaldo Cruz, FIOCRUZ, Rio de Janeiro, RJ, Brazil.

^2^ Laboratório de Comportamento de Insetos, Instituto Federal do Norte de Minas Gerais, Salinas, MG, Brazil.

^3^ Laboratório de Diptera, Instituto Oswaldo Cruz, FIOCRUZ, Rio de Janeiro, RJ, Brazil.

^4^ Programa de Pós-Graduação em Biologia Parasitária, Universidade Federal de Sergipe, São Cristóvão, SE, Brazil.

^5^ Instituto Pasteur, São Paulo, SP, Brazil.

^#^ Filipe Vieira Santos de Abreu and Lucas Bonato Mosmann contributed equally to this work.

* Correspondence: [lourenco@ioc.fiocruz.br](mailto:lourenco@ioc.fiocruz.br), [mgpavan@ioc.fiocruz.br](mailto:mgpavan@ioc.fiocruz.br)


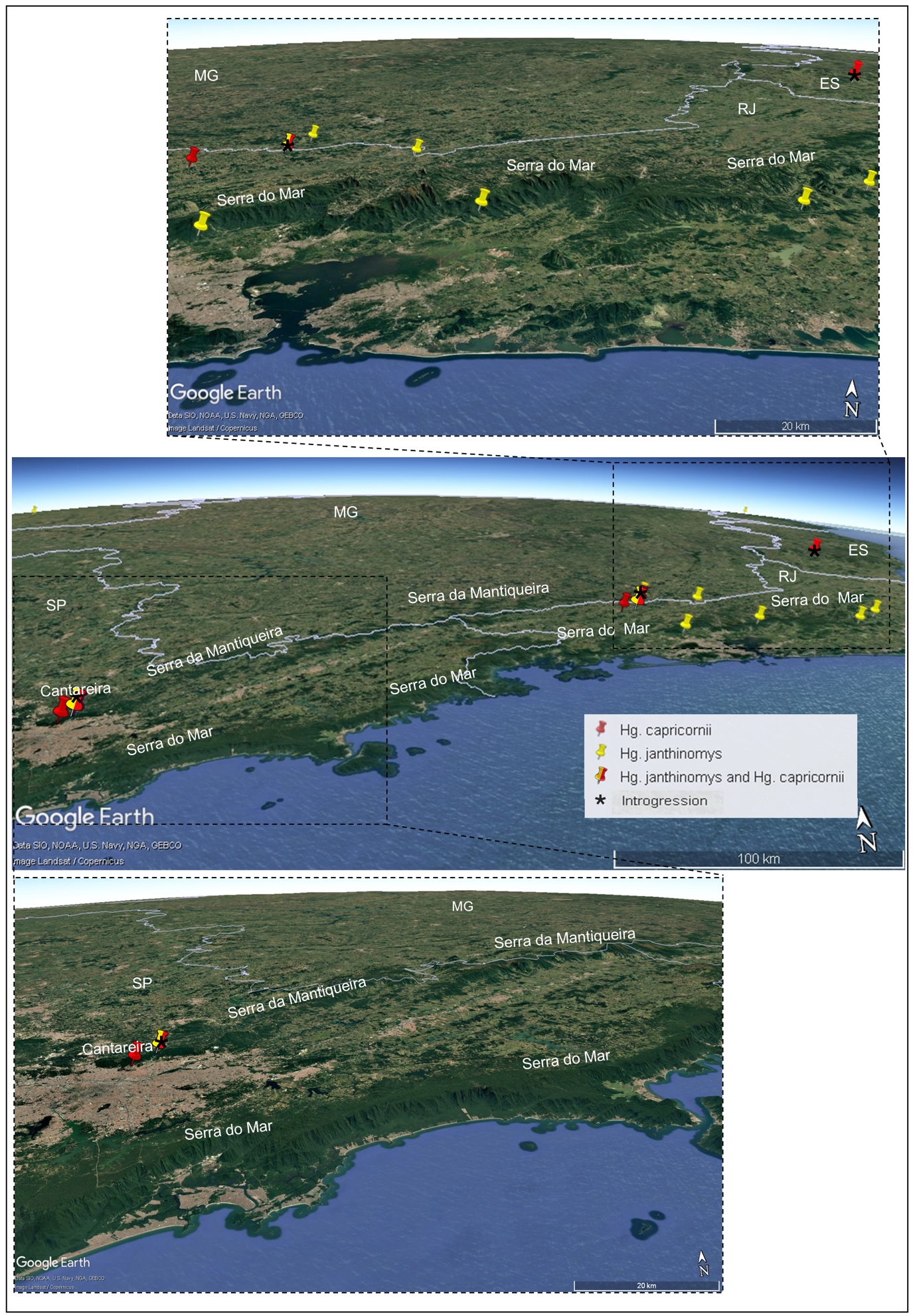


**Figure S1**. Satellite map showing the collection sites of *Hg. janthinomys* (yellow pins) and *Hg. capricornii* (red pins) in the Brazilian states of Espírito Santo (ES), Minas Gerais (MG), Rio de Janeiro (RJ), and São Paulo (SP). In these images, it is possible to observe the main mountain ranges of the Serra do Mar and Serra da Mantiqueira. Areas of *Hg. janthinomys* and *Hg. capricornii* co-occurrence were shown with half yellow and half red pins.

**
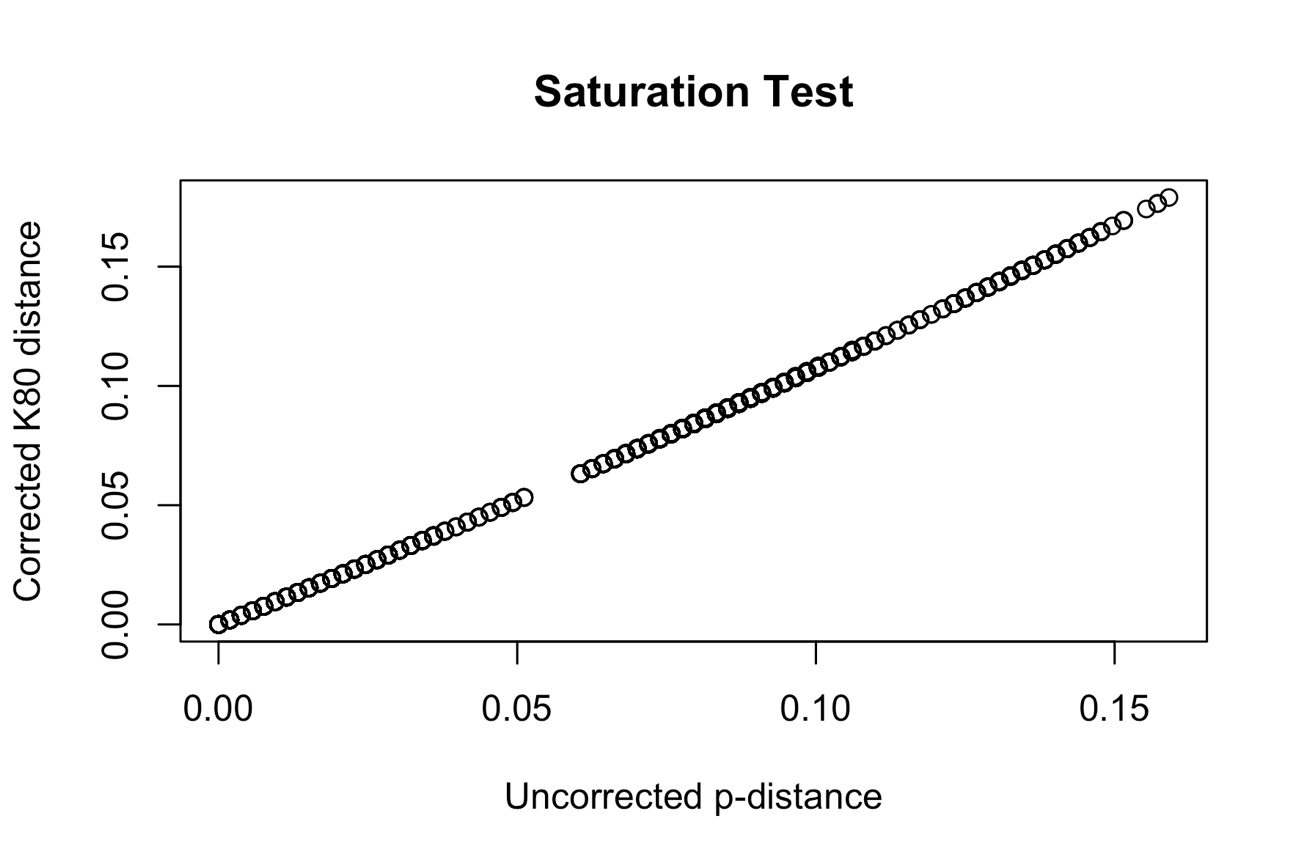
**

**Figure S2. Saturation plot with COI sequences using uncorrected pairwise p-distance values (x-axis) and corrected pairwise K2-P (or K80) distances (y-axis).** When saturated, a plateau would be observed in the plotted distances, which did not occur in our dataset.


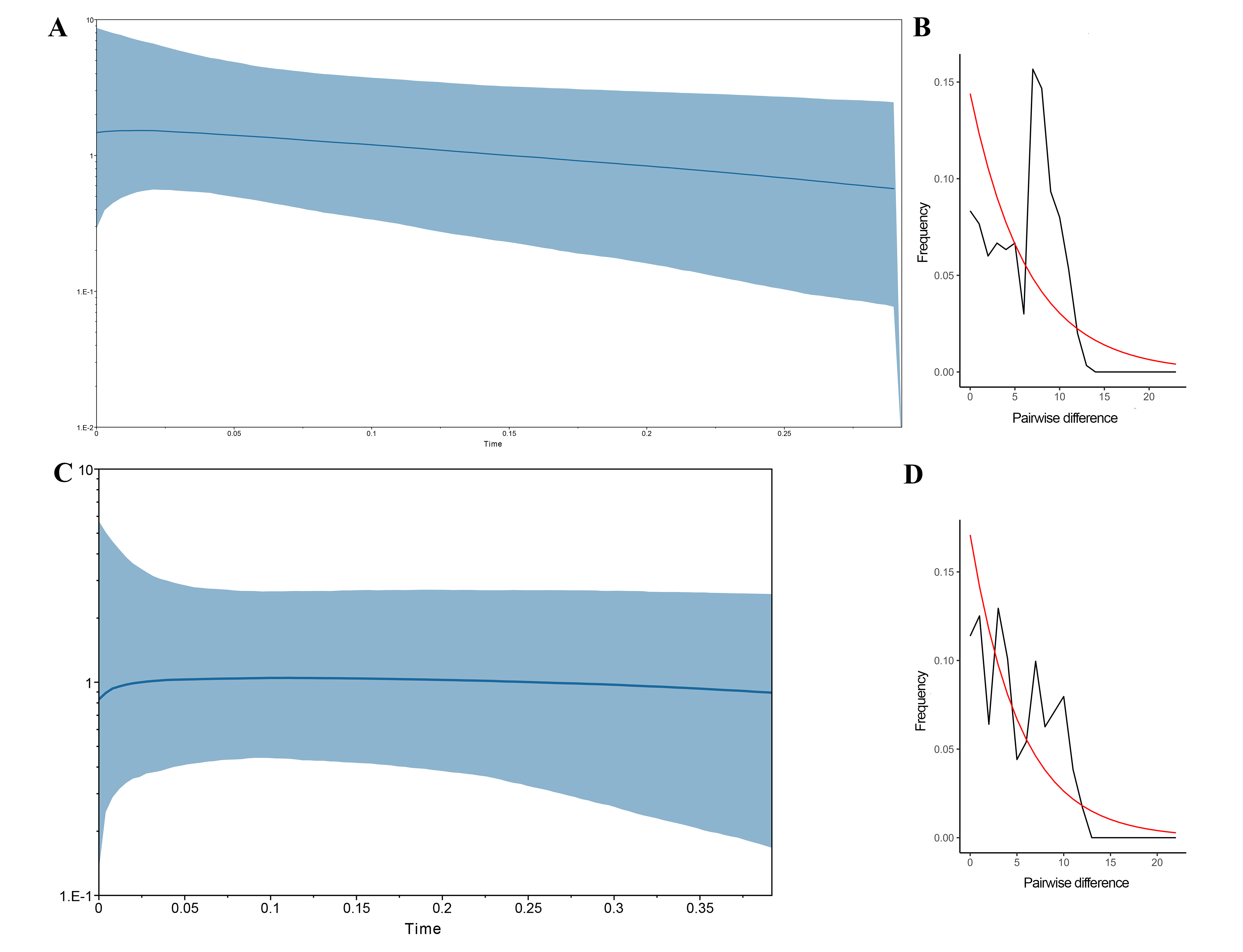


**Figure S3: Population analyses based on *Hg. janthinomys* mt-COI sequences.**(A) Bayesian Skyline Plot (BSP) of the first *Hg. janthinomys* subclade. The blue line shows changes in effective population size over time, while the shaded area represents the 95% highest posterior density interval. The X-axis represents time in millions of years before present, and the Y-axis corresponds to relative effective population size. (B) Mismatch distribution of the first *Hg. janthinomys* subclade. The black line represents the observed distribution of pairwise nucleotide differences, and the red line indicates the expected distribution under a model of sudden demographic expansion. (C) Bayesian Skyline Plot (BSP) of the second *Hg. janthinomys* subclade. (D) Mismatch distribution of the second *Hg. janthinomys* subclade.
